# Albuminuria, kidney function, and automatically quantified retinal vascular phenotypes in hospitalized adults with type 2 diabetes

**DOI:** 10.3389/fendo.2026.1957415

**Published:** 2026-09-28

**Authors:** Hongyun Wu, Likun Yu, Wei Ye, Xi Wang, Zhe Xu

**Affiliations:** 1 Ganzhou Key Laboratory of Ocular Trauma, Department of Ophthalmology, Ganzhou Hospital–Nanfang Hospital, Southern Medical University (Ganzhou People’s Hospital), Ganzhou, Jiangxi, China; 2 The Affiliated Ganzhou Hospital, Jiangxi Medical College, Nanchang University, Ganzhou, Jiangxi, China; 3 EVision Technology (Beijing) Co., Ltd., Beijing, China

**Keywords:** albuminuria, artificial intelligence, estimated glomerular filtration rate, retinal vasculature, type 2 diabetes

## Abstract

**Objective:**

To compare albuminuria and creatinine-based estimated glomerular filtration rate (eGFR) in relation to automatically quantified retinal vascular phenotypes from color fundus photographs in hospitalized adults with type 2 diabetes.

**Methods:**

This single-center retrospective study applied clinical, ocular, image-quality, and temporal eligibility criteria. Kidney measurements with a verifiable laboratory-report-to-image interval of no more than 7 days were retained. Exact urinary albumin-to-creatinine ratio (UACR) values were analyzed after log2 transformation; eGFR was recalculated using the 2021 Chronic Kidney Disease Epidemiology Collaboration (CKD-EPI) creatinine equation. Eight vascular outcomes were analyzed at the eye level using patient-clustered robust standard errors. The prespecified primary analyses were separate core models for UACR and eGFR adjusted for age, gender, body mass index, diabetes duration, HbA1c, and systolic blood pressure, with Benjamini–Hochberg false discovery rate (FDR) correction across the eight outcomes within each exposure family. Joint, complete-case, alternative-equation, restricted-range, categorical, spline, and tighter-temporal-window analyses were treated as supportive or exploratory.

**Results:**

The final cohort included 658 eyes from 337 patients; the median absolute laboratory-report-to-image interval was 1 day (IQR, 0–1), and 97.9% of eye records were within 3 days. In the primary separate core models, neither UACR nor 2021 CKD-EPI eGFR was associated with any of the eight vascular outcomes after FDR correction (minimum *q* = 0.090). UACR showed a nominal association with arterial density (*q* = 0.097), and eGFR showed nominal associations with overall, arterial, and 3-mm foveal vessel density. Exploratory complete-case analyses yielded stronger eGFR signals: in the 431-eye/220-patient joint complete-case cohort, the eGFR-only model in the same cohort was FDR-significant for all eight outcomes, and seven remained significant after mutual adjustment with UACR. Alternative-equation and restricted-range analyses also yielded eGFR-related associations, whereas same-day analyses were imprecise and null. In a *post hoc* smoking-adjusted joint model, however, none of the eight eGFR associations remained significant after FDR correction.

**Conclusions:**

The primary analyses did not demonstrate FDR-significant associations of either kidney marker with the retinal vascular phenotypes. Exploratory complete-case and sensitivity analyses suggested a model-dependent eGFR-related pattern, whereas UACR remained largely null. These cross-sectional findings are hypothesis-generating and require independent longitudinal validation.

## Introduction

Diabetic kidney disease and diabetic retinopathy are common microvascular complications of type 2 diabetes and remain major causes of kidney failure and visual impairment, respectively (1–8). Kidney involvement is assessed primarily using the urinary albumin-to-creatinine ratio (UACR) and estimated glomerular filtration rate (eGFR). These measures provide complementary rather than interchangeable information. UACR reflects increased glomerular permeability and urinary albumin excretion, whereas eGFR estimates filtration capacity. Albuminuria may occur while eGFR remains preserved, and kidney function may decline without substantial albuminuria (1–3, 9, 10).

Clinical diabetic retinopathy grading is based mainly on characteristic lesions and their severity (4–8). Color fundus photographs also permit quantitative assessment of the visible retinal vascular network. Vessel density describes the proportion of an analyzed retinal region occupied by detectable vessels, whereas fractal dimension summarizes the geometric complexity of vascular branching (11). These continuous measurements characterize aspects of retinal vascular architecture that are not directly represented by conventional retinopathy categories. Shared endothelial injury and microvascular remodeling provide a biological rationale for examining retinal vascular structure in relation to kidney disease, and previous reviews have summarized associations between retinal vascular abnormalities and chronic kidney disease (12, 13).

Observational studies have examined retinal vascular caliber, density, fractal geometry, and optical coherence tomography angiography (OCTA)-derived perfusion measures in relation to albuminuria, eGFR, or clinically defined kidney disease, with inconsistent results (14–22). Automated analysis of color fundus photographs has linked kidney disease to retinal caliber, arteriolar tortuosity, image-texture measures, vessel density, and fractal dimension, although the features retained after multivariable adjustment have differed between studies (20, 22). An OCTA study reported lower retinal capillary density in association with lower eGFR and higher albuminuria (21). However, vessel density from segmentation of visible vessels on a color fundus photograph is technically and physiologically distinct from OCTA-derived capillary perfusion, and the two measures should not be interpreted as equivalent. Across population-based and diabetes-specific studies, the direction and strength of observed associations have varied after adjustment for clinical covariates (14–19), potentially reflecting differences in populations, imaging modalities, analysis regions, definitions of kidney disease, covariate selection, and handling of bilateral-eye data.

Retinal photographs have also been used in deep-learning models to detect prevalent CKD and estimate future CKD risk (23–26). More recently, DeepDKD used retinal photographs to detect diabetic kidney disease and distinguish isolated diabetic nephropathy from nondiabetic kidney disease, whereas the Kidney Intelligent Diagnosis System (KIDS) framework combined retinal information with clinical data for CKD screening and pathological-type identification (34, 35). These prediction and pathology-classification studies demonstrate that kidney-related information can be extracted from retinal images, but they address a different question from the present analysis: they do not by themselves establish which interpretable vascular measurements are associated with contemporaneous albuminuria or filtration estimates.

We therefore examined UACR and creatinine-based eGFR as distinct exposures in relation to eight predefined, automatically quantified retinal vascular phenotypes from color fundus photographs in hospitalized adults with type 2 diabetes. The primary objective was to compare their separate associations after adjustment for core clinical covariates. Joint and sensitivity analyses were undertaken as supportive or exploratory analyses to assess whether findings depended on complete-case cohort composition, covariate specification, temporal matching, the eGFR equation, or the upper eGFR range. The study was not designed to diagnose CKD or diabetic kidney disease, nor to evaluate prediction, discrimination, calibration, or incremental clinical utility.

## Materials and methods

### Study design and population

This single-center retrospective observational study was conducted at Ganzhou People’s Hospital. The source workbook comprised inpatient records from the Department of Endocrinology with retinal imaging obtained between 24 October 2023 and 11 March 2025. The audited source dataset contained 1,058 populated eye-level records from 529 patients.

Patients were eligible if they had type 2 diabetes, were 18–80 years of age, and had at least one eligible color fundus photograph. The preexisting curated dataset excluded type 1 diabetes, acute systemic inflammation, malignant tumor, and images failing quality assessment because of blur, inadequate illumination, media opacity, or incomplete visualization. Eye-level exclusions included a history of glaucoma, vitrectomy, or retinal laser treatment and fundus findings likely to distort quantitative vascular measurements, including severe fundus tessellation, optic atrophy, epiretinal membrane, and macular hole. Severe fundus tessellation was initially flagged by EVisionAI and confirmed by ophthalmologist review before exclusion.

Records were linked by hospitalization identifier, admission date, eye, and image identifier. The laboratory-report date and retinal image date were used to calculate the absolute laboratory-report-to-image interval. Records with an unavailable interval or an interval greater than 7 days were excluded. For each matched hospitalization, the laboratory result closest to retinal imaging was retained. Availability of UACR or serum creatinine was required only for the corresponding kidney-marker analysis and not for inclusion in the final retinal imaging cohort.

### Clinical and biochemical data collection

Age, gender, body mass index (BMI), diabetes duration, blood pressure, hemoglobin A1c (glycated hemoglobin) (HbA1c), medication history, smoking history, serum creatinine, UACR, and other clinical data were extracted from the inpatient information system. Smoking history and documented antihypertensive treatment were available as structured binary fields in the analytical workbook, whereas lipid measurements were available for a subset, and a structured cardiovascular-comorbidity variable was not available for modeling. Diabetes duration was harmonized from the original records and expressed in years. Medication variables represented preadmission use explicitly documented in the history of present illness or past medical history. Absence of documentation was coded as no documented use; inpatient orders and pharmacy dispensing data were not used.

Urinary microalbumin was measured by immunoturbidimetry on a Beckman Coulter IMMAGE 800 specific protein analyzer, and urinary creatinine by the sarcosine oxidase method on a Beckman Coulter AU5800 analyzer. The analyzers, reagents, measurement methods, calculation formula, and reporting rules remained unchanged during the study period. The study laboratory confirmed a local urinary microalbumin reportable range of 0–1,800 mg/dL. UACR (mg/g) was calculated in the laboratory information system as MA × 10,000/UCRE/0.113, where MA is urinary microalbumin in milligrams per deciliter, and urinary creatinine (UCRE) is urinary creatinine in μmol/L. UACR was categorized as < 30, 30–299, or ≥ 300 mg/g. Additional assay details, calculation formulas, and reporting rules are summarized in **Supplementary Table S2**.

Most UACR results were reported as exact numeric values. When urinary microalbumin was outside the assay reportable range, the laboratory information system returned a record-specific inequality value (< *x* or > *x*). Continuous analyses excluded these observations because their exact values could not be recovered. Categorical analyses retained an inequality-reported value only when its UACR category was unambiguous; a boundary crossing a category threshold was treated as unclassifiable.

Serum creatinine was measured on a Beckman Coulter AU5800 analyzer using the Creatinine (Enzymatic) OSR61204 assay based on the creatininase-creatinase-sarcosine oxidase reaction. The serum calibrator was traceable through NIST SRM 967 to an isotope-dilution mass spectrometry reference measurement procedure. Creatinine was converted from micromoles per liter to milligrams per deciliter by division by 88.4. The primary eGFR exposure was recalculated using the 2021 CKD-EPI creatinine equation without a race coefficient (27). The laboratory-reported Chinese-modified abbreviated Modification of Diet in Renal Disease (MDRD) estimate was examined in sensitivity analyses (28).

### Retinal image acquisition, grading, and quantitative analysis

Color fundus photographs were acquired without pharmacological dilation using a Canon CR-2 nonmydriatic camera (Canon Inc., Tokyo, Japan), with a 45°field of view and 3,024 pixel × 3,024 pixel resolution. Images were processed with EVisionAI version 4.0 (Evision Technology [Beijing] Co. Ltd., Beijing, China). The automated pipeline included retinal-region extraction, image normalization, quality assessment, vessel segmentation, artery-vein classification, lesion detection, and foveal localization. Published work using related automated retinal-quantification frameworks has described ResNet101-UNet vessel segmentation (36). A separate 2026 study explicitly using the EVisionAI system reported a TransUNet-based segmentation pipeline, multicenter annotated development data, ophthalmologist annotation with semiautomatic refinement, vessel-segmentation intersection-over-union > 0.99, and optic-disc intersection-over-union of 0.939 (37). These reports support the broader technical approach but do not constitute version-specific validation of every EVisionAI 4.0 output used in the present cohort. In particular, artery–vein classification accuracy, quantitative foveal-localization error, and test–retest reproducibility of vessel-density and fractal-dimension measures were not independently assessed in this cohort. **Figure 1** therefore presents representative qualitative outputs rather than validation performance.

**Figure 1:**
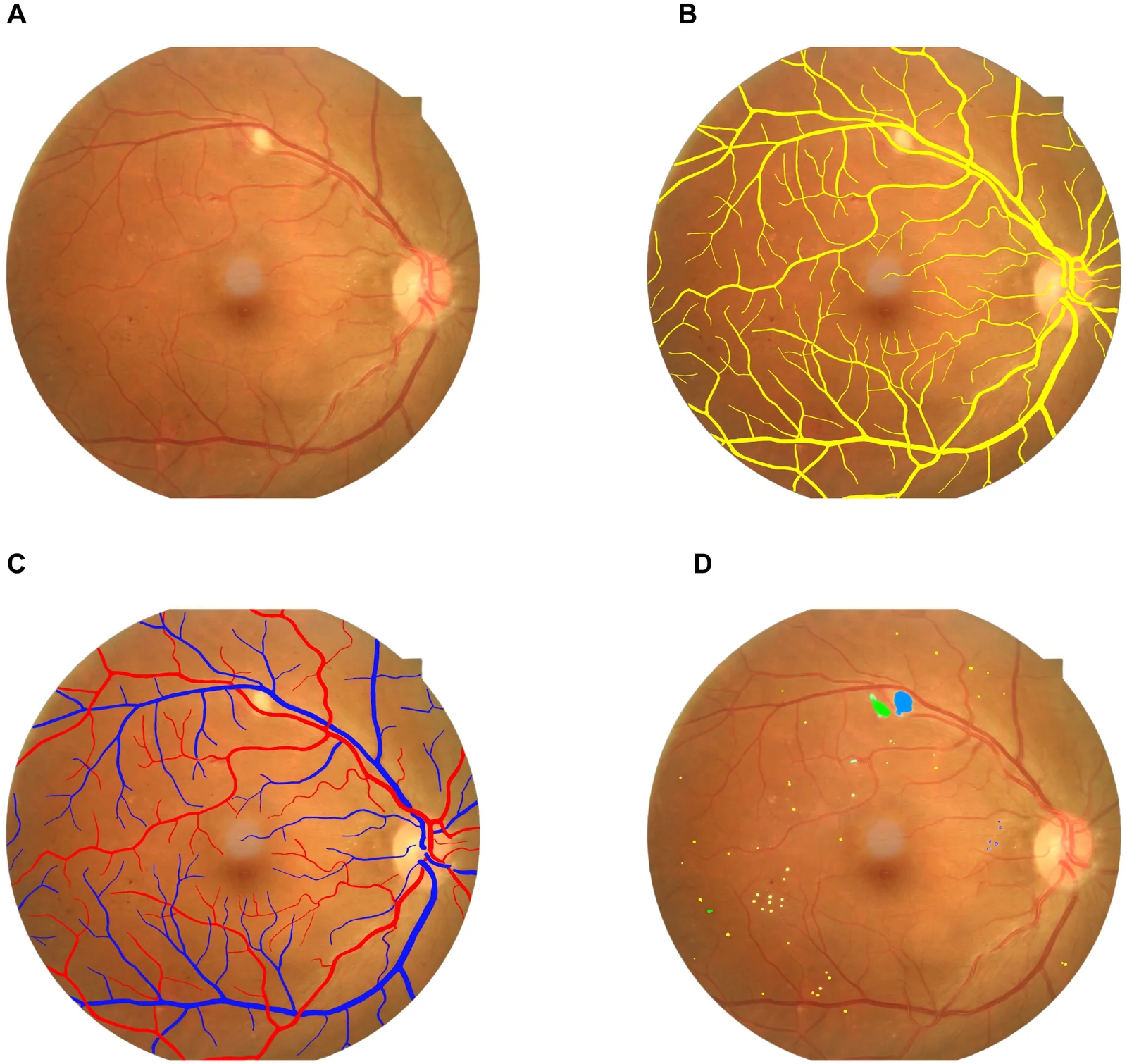
Representative fundus photograph and automatically quantified EVisionAI image-analysis outputs. All panels were derived from the same representative eye and provide a qualitative illustration of the image-analysis workflow; they do not represent algorithm-validation or reproducibility performance. **(A)** Original color fundus photograph. **(B)** Automated vessel segmentation. **(C)** Artery–vein classification (red, arteries; blue, veins). **(D)** Lesion detection (yellow, microaneurysms; green, hemorrhages; blue, hard exudates; light blue, soft exudates).

Before quantitative analysis, EVisionAI version 4.0 performed automated image-quality control. The quality-control workflow first classified each input to determine whether it was a fundus photograph and excluded non-fundus images. The platform then localized the optic disc and macula and assessed image clarity using a score ranging from 0 to 1, derived primarily from retinal-vessel line extraction and the extent of detectable vascular coverage. Failure of any of these components resulted in the image being classified as unsuitable for quantitative analysis. The exact proprietary numerical cutoff applied to the 0–1 clarity score was not available in the exported study documentation. For all 658 eyes in the final analytical cohort, retinal vessel segmentation, artery–vein classification, foveal-center localization, and calculation of the final quantitative vascular parameters were performed automatically by EVisionAI version 4.0; no vessel masks, artery–vein labels, foveal locations, or derived vascular measurements were manually edited or corrected before statistical analysis (0 of 658 eyes underwent manual modification). According to a retrospective audit of the EVisionAI platform logs, eight of the 872 eyes (0.9%) remaining after the initial clinical, ocular, and image-quality exclusions had two or more quality-eligible candidate photographs. For these eyes, the platform analyzed the eligible photographs and retained the image associated with the highest automatically assessed diabetic retinopathy severity.

The software used fixed metric-specific analysis masks, numerators, and denominators. Overall vessel density was computed within the full predefined retinal region and included the optic disc. For measurements designated as non-disc regional metrics, the optic disc was excluded from both the numerator and denominator. The foveal center was localized automatically, and mean vessel density was calculated within circles of 3- and 5-mm diameter centered on the detected fovea. Lesion areas were converted from pixels to square millimeters using image-specific pixel-pitch calibration (29). This calibration standardizes camera/image scaling but cannot fully correct patient-specific ocular magnification related to axial length or refractive error, which were unavailable in the present dataset.

Vascular fractal dimension was calculated from the binary vessel segmentation map using a multiscale box-counting procedure. At each box size *ϵ*, *N*(*ϵ*) was the number of boxes containing at least one vessel pixel. Fractal dimension was estimated as the slope from linear regression of ln[*N*(*ϵ*)] on ln(1/*ϵ*) across the predefined scales. The procedure was applied to the overall, arterial, and venous binary maps. Detailed operational definitions are provided in **Supplementary Table S13** and **Supplementary Figure S1**.

Diabetic retinopathy severity was graded separately for each eye by a single ophthalmologist who was masked to the corresponding UACR and eGFR results, according to the International Clinical Diabetic Retinopathy Disease Severity Scale (7). For statistical analysis, severity was coded as 0 for no apparent diabetic retinopathy, 1 for mild non-proliferative diabetic retinopathy, 2 for moderate non-proliferative diabetic retinopathy, 3 for severe non-proliferative diabetic retinopathy, and 4 for proliferative diabetic retinopathy. As grading was performed by a single masked ophthalmologist, intergrader reproducibility was not assessed.

### Statistical analysis

Analyses were performed with Python 3.13.5, NumPy 2.3.5, SciPy 1.17.0, and statsmodels 0.14.6. Continuous variables are summarized as median (interquartile range), and categorical variables as *n*/*N* (%). Baseline comparisons were performed at the patient level, with the worse-eye diabetic retinopathy stage used for patient-level summaries. Kruskal–Wallis, Chi-square, or Fisher exact tests were used as appropriate. Eye-level phenotype tables are descriptive and omit conventional *p*-values because fellow-eye observations are correlated.

The inferential outcome family comprised eight *a priori* selected vascular measures: overall, arterial, and venous vessel density; mean vessel density in the 3- and 5-mm foveal regions; and overall, arterial, and venous fractal dimension. Exact numeric UACR values were log2-transformed, so the coefficient represents the mean outcome difference associated with each doubling of UACR. eGFR was coded per 10 mL/min/1.73 m^2^ decrease; negative coefficients therefore indicate lower retinal measures with lower kidney function.

The primary core model adjusted for age, gender, BMI, diabetes duration, HbA1c, and systolic blood pressure. Eye-level ordinary least-squares regression used patient-clustered robust standard errors with finite-sample correction. A medication-expanded model additionally adjusted for documented pre-admission insulin, ACEI/ARB, and statin use. A further sensitivity model added the diabetic retinopathy stage of the corresponding eye. Joint models entered log2-transformed UACR and eGFR simultaneously. To distinguish complete-case cohort composition from effects of mutual adjustment, the UACR- and eGFR-only core models were additionally refitted in the same complete-case cohort used for the joint model. Generalized estimating equations used a Gaussian family, identity link, exchangeable working correlation, and robust sandwich variance. After a raw-data audit showed that smoking history was complete in the final cohort but differently distributed between patients included in and those excluded from the joint complete-case analysis, additional models adjusted for documented smoking history. These smoking-adjusted analyses were *post hoc* and were interpreted as exploratory rather than as replacements for the prespecified core models.

Sensitivity analyses used the laboratory-reported Chinese-modified MDRD eGFR, restricted the CKD-EPI analysis to eGFR < 120 mL/min/1.73 m^2^, modeled eGFR categories (< 90, 90–119, and ≥ 120 mL/min/1.73 m^2^), additionally adjusted for the laboratory-report-to-image interval, and restricted the temporal window to ≤ 3 days or the same laboratory-report and image date. The eGFR categories (< 90, 90–119, and ≥ 120 mL/min/1.73 m^2^) and the complementary restriction to eGFR < 120 mL/min/1.73 m^2^ were specified *a priori*, before inspection of the study eGFR distribution or outcome results. KDIGO classifies eGFR ≥ 90 mL/min/1.73 m^2^ as G1 (normal or high in the absence of other CKD-defining abnormalities) but does not subdivide values within G1 (2). We therefore prespecified 90–119 mL/min/1.73 m^2^ as the reference stratum and ≥ 120 mL/min/1.73 m^2^ as a separate high-eGFR stratum. This was an analytical choice intended to distinguish the 90–119 portion of G1 from values in a range that has been used operationally in hyperfiltration research. In type 2 diabetes, measured GFR ≥ 120 mL/min/1.73 m^2^ and eGFR > 120 mL/min/1.73 m^2^ have both been used to define hyperfiltration (38, 39); an epidemiologic study also analyzed eGFR > 120 and 90–119 mL/min/1.73 m^2^ as separate strata, although its formal hyperfiltration definition was age- and gender-specific (40). Hyperfiltration is not uniformly defined and may depend on age, gender, population, and GFR measurement or estimating method; we did not treat ≥ 120 mL/min/1.73 m^2^ as a diagnostic cutoff. The eGFR < 120 analysis was the complementary prespecified restricted-range analysis. These cut points were not intended to define reduced kidney function or CKD. Restricted cubic splines with knots at the fifth, 35th, 65th, and 95th percentiles assessed nonlinearity. Standardized coefficients were calculated as β × SD(exposure)/SD(outcome) within each model-specific complete-case sample. Missingness and complete-case selection were described with standardized mean differences. In addition to the existing kidney-marker availability comparisons, the 220 patients entering the joint core complete-case analysis were directly compared with the remaining 117 patients in the final retinal cohort (**Supplementary Table S12E**).

No formal *a priori* sample-size calculation was performed because this was a retrospective study; all available records meeting the eligibility criteria were included.

All tests were two-sided. Benjamini–Hochberg correction controlled the FDR across the eight outcomes separately within each exposure-model family (30). For categorical eGFR analyses, FDR correction was applied separately across the eight outcomes for each contrast (< 90 vs. 90–119 and ≥ 120 vs. 90–119 mL/min/1.73 m^2^). *q* < 0.05 was considered statistically significant after multiplicity correction. FDR was controlled within individual model families rather than across all analytical choices; therefore, results from joint, same-cohort complete-case, alternative-equation, restricted-range, categorical, spline, tighter-temporal-window, and *post hoc* smoking-adjusted analyses were interpreted as supportive or exploratory and hypothesis-generating rather than as replacements for the primary separate core models. Reporting followed the STROBE statement (31).

### Ethics statement

The study conformed to the Declaration of Helsinki and was approved by the Ethics Committee of Ganzhou People’s Hospital (Approval Number B2026-176-01). The requirement for written informed consent was waived because of the retrospective design and use of de-identified data.

## Results

### Study population and temporal matching

The source worksheet contained 1,058 populated eye-level records from 529 patients. After the previously applied clinical, ocular, and image-quality exclusions, 872 eyes from 450 patients remained. Age restriction removed 11 eyes from six patients. The laboratory-report-to-image interval was unavailable for 111 eyes from 57 patients; among records with a verifiable interval, 90 eyes from 48 patients exceeded 7 days. Two of 660 eyes (0.3%) satisfying the temporal criterion had invalid or incomplete values for the eight core vascular outputs and were excluded. The final retinal imaging cohort therefore comprised 658 eyes from 337 patients, including 321 patients with bilateral-eye data and 16 with unilateral-eye data (**Figure 2**; **Supplementary Table S1**).

**Figure 2:**
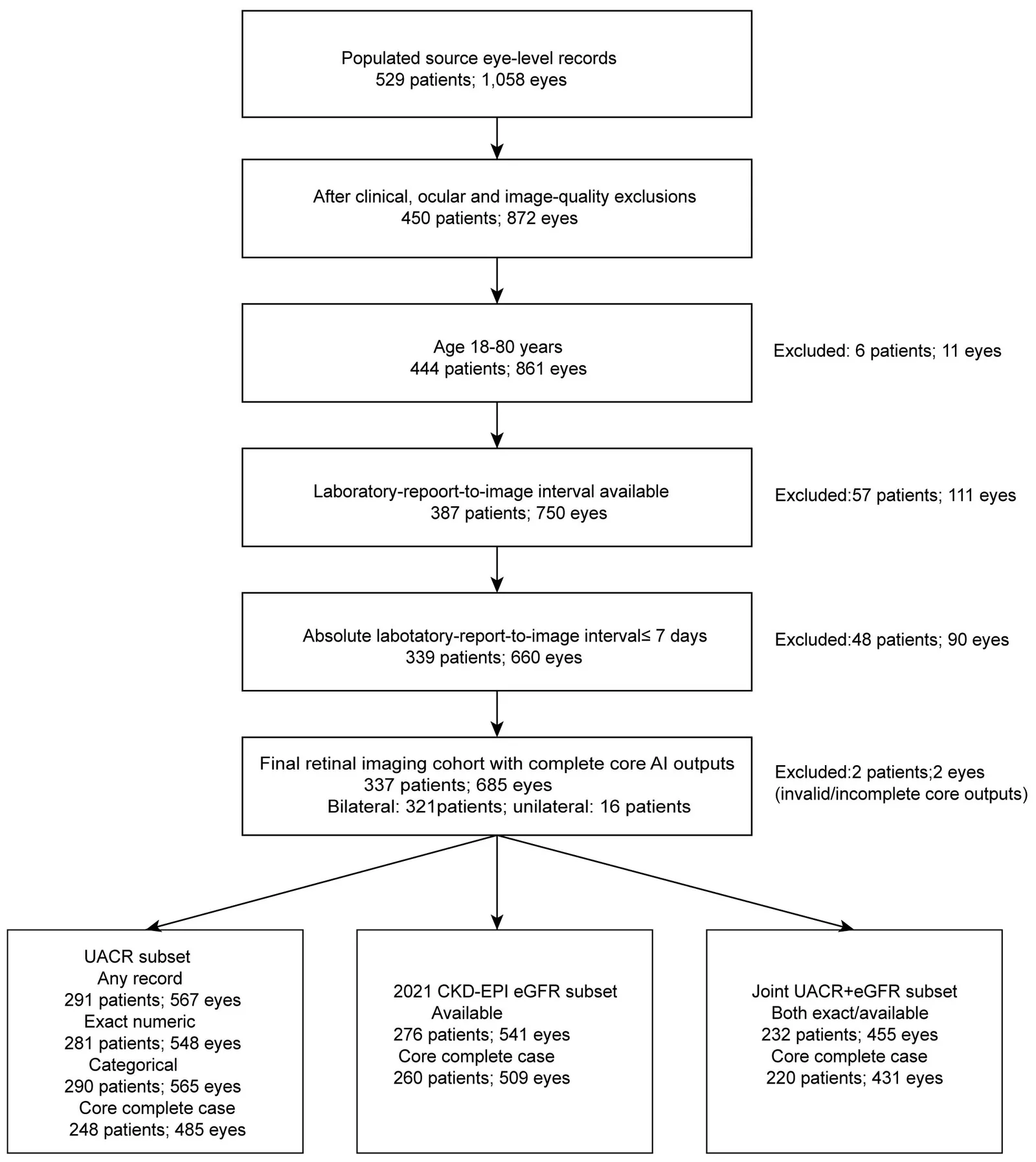
Study flow diagram. Kidney-marker subsets overlap and are not mutually exclusive. Continuous UACR analyses used exact numeric values only. The median absolute laboratory-report-to-image interval in the final cohort was 1 day (IQR, 0–1); 97.9% of eye records fell within 3 days.

The median absolute laboratory-report-to-image interval was 1 day (interquartile range IQR, 0–1) at both the eye and patient levels. At the eye level, 280/658 records (42.6%) had identical laboratory-report and image dates, 534/658 (81.2%) were within 1 day, and 644/658 (97.9%) were within 3 days; the maximum interval was 6 days. Any UACR result was available for 567 eyes from 291 patients. Nineteen eyes from 10 patients had inequality-reported UACR, leaving 548 eyes from 281 patients for continuous analysis and 565 eyes from 290 patients for categorical analysis. Recalculated CKD-EPI eGFR was available for 541 eyes from 276 patients; 455 eyes from 232 patients had both exact UACR and eGFR.

Among 224 unique patients with both eGFR estimates available, the median 2021 CKD-EPI eGFR was 103.7 mL/min/1.73 m^2^ (IQR, 93.2–112.3), compared with 132.7 mL/min/1.73 m^2^ (IQR, 108.7–160.9) for the laboratory-reported Chinese-modified MDRD estimate. The median paired difference was 29.5 mL/min/1.73 m^2^ (IQR, 14.2–50.1), and the two estimates were strongly correlated (Spearman *ρ* = 0.875; *p* < 0.001; **Supplementary Table S3**).

### Baseline characteristics

The categorical UACR analysis included 201 patients with UACR < 30 mg/g, 69 with UACR 30–299 mg/g, and 20 with UACR ≥ 300 mg/g (**Table 1**). Higher categories were associated with longer diabetes duration, higher systolic blood pressure, higher serum creatinine, lower CKD-EPI eGFR, and more advanced diabetic retinopathy. Nine inequality-reported values were assigned unambiguously to a category; one result reported as < 30.46 mg/g was excluded from categorical analysis because its boundary crossed the 30 mg/g threshold.

**Table 1:** Baseline characteristics according to UACR category.

Characteristic	Overall (*n* = 290)	< 30 mg/g (*n* = 201)	30–299 mg/g (*n* = 69)	≥ 300 mg/g (*n* = 20)	*p*-value
Age (years)	55.0 (46.2, 64.0)	55.0 (46.0, 62.0)	57.0 (48.0, 66.0)	64.0 (48.8, 66.0)	0.127
Female sex (*n*, %)	84/290 (29.0)	56/201 (27.9)	20/69 (29.0)	8/20 (40.0)	0.521
BMI (kg/m^2^)	23.53 (21.38, 25.66)	23.34 (21.27, 25.16)	23.88 (21.78, 27.00)	24.14 (21.29, 26.52)	0.247
Diabetes duration (years)	3.0 (0.3, 10.0)	2.0 (0.2, 8.0)	3.0 (0.5, 10.0)	8.0 (3.8, 10.0)	0.017
Systolic blood pressure (mmHg)	131.0 (122.0, 142.0)	129.0 (119.0, 140.0)	135.0 (125.0, 147.0)	144.5 (125.8, 164.2)	0.009
Diastolic blood pressure (mmHg)	86.0 (78.0, 94.0)	84.0 (78.0, 93.0)	88.0 (80.0, 96.0)	88.0 (73.0, 93.5)	0.383
HbA1c (%)	10.85 (8.20, 12.40)	11.00 (7.95, 12.35)	10.80 (8.90, 12.70)	10.85 (8.95, 11.67)	0.954
Serum creatinine (μmol/L)	65.0 (54.0, 77.0)	64.0 (54.0, 74.2)	65.0 (53.3, 80.2)	91.6 (73.0, 113.5)	< 0.001
2021 CKD-EPI eGFR (mL/min/1.73 m^2^)	104.7 (93.7, 114.3)	106.6 (97.8, 115.0)	103.9 (91.8, 111.9)	80.7 (63.8, 94.7)	< 0.001
Diabetic retinopathy stage (worse eye)	0.0 (0.0, 1.0)	0.0 (0.0, 1.0)	0.0 (0.0, 1.0)	1.0 (0.0, 2.0)	< 0.001
Any diabetic retinopathy (*n*, %)	105/290 (36.2)	59/201 (29.4)	32/69 (46.4)	14/20 (70.0)	< 0.001
Documented preadmission insulin use (*n*, %)	73/290 (25.2)	46/201 (22.9)	18/69 (26.1)	9/20 (45.0)	0.092
Documented preadmission ACEI/ARB use (*n*, %)	15/290 (5.2)	10/201 (5.0)	2/69 (2.9)	3/20 (15.0)	0.096
Documented preadmission statin use (*n*, %)	28/290 (9.7)	17/201 (8.5)	8/69 (11.6)	3/20 (15.0)	0.527
Documented pre-admission beta-blocker use (*n*, %)	11/290 (3.8)	7/201 (3.5)	3/69 (4.3)	1/20 (5.0)	0.909

Values are median (IQR) unless otherwise indicated. Denominators vary because of missing values. UACR categories retained inequality-reported values only when category assignment was unambiguous. Diabetic retinopathy stage was defined by the worst eligible eye. *p*-values were calculated at the patient level. Medication variables indicate documented preadmission use; absence of documentation was coded as no documented use.

The eGFR analysis included 13 patients with eGFR < 60 mL/min/1.73 m^2^, 39 with eGFR 60–89 mL/min/1.73 m^2^, and 224 with eGFR ≥ 90 mL/min/1.73 m^2^ (**Table 2**). Lower eGFR categories were associated with older age, longer diabetes duration, higher UACR, more advanced diabetic retinopathy, and more frequent documented insulin, statin, and beta-blocker use. The small number with eGFR < 60 limits precision of category-specific comparisons.

**Table 2:** Baseline characteristics according to 2021 CKD-EPI eGFR category.

Characteristic	Overall (*n* = 276)	< 60 (*n* = 13)	60–89 (*n* = 39)	≥ 90 (*n* = 224)	*p*-value
Age (years)	55.0 (46.0, 63.0)	68.0 (59.0, 71.0)	62.0 (52.5, 67.0)	54.0 (44.0, 61.0)	< 0.001
Female sex (*n*, %)	80/276 (29.0)	4/13 (30.8)	9/39 (23.1)	67/224 (29.9)	0.679
BMI (kg/m^2^)	23.56 (21.53, 25.70)	25.53 (23.56, 28.20)	22.80 (20.97, 24.37)	23.62 (21.59, 25.79)	0.048
Diabetes duration (years)	3.0 (0.5, 10.0)	10.0 (6.0, 12.0)	6.0 (0.3, 10.0)	2.0 (0.5, 8.0)	0.009
Systolic blood pressure (mmHg)	131.0 (120.0, 142.0)	131.0 (128.0, 141.0)	131.0 (126.0, 144.0)	131.0 (118.8, 142.0)	0.739
Diastolic blood pressure (mmHg)	85.0 (78.0, 93.0)	80.0 (76.0, 92.0)	88.0 (80.5, 94.0)	84.0 (78.0, 93.0)	0.459
HbA1c (%)	10.80 (8.40, 12.40)	10.40 (8.12, 13.48)	10.05 (8.25, 12.65)	10.90 (8.53, 12.28)	0.790
Exact numeric UACR (mg/g)	15.360 (8.277, 41.460)	29.340 (20.580, 443.455)	61.120 (12.180, 230.040)	12.960 (7.870, 30.400)	< 0.001
Diabetic retinopathy stage (worse eye)	0.0 (0.0, 1.0)	2.0 (0.0, 2.0)	0.0 (0.0, 2.0)	0.0 (0.0, 1.0)	0.013
Any diabetic retinopathy (*n*, %)	103/276 (37.3)	8/13 (61.5)	19/39 (48.7)	76/224 (33.9)	0.038
Documented preadmission insulin use (*n*, %)	68/276 (24.6)	7/13 (53.8)	6/39 (15.4)	55/224 (24.6)	0.021
Documented preadmission ACEI/ARB use (*n*, %)	14/276 (5.1)	2/13 (15.4)	2/39 (5.1)	10/224 (4.5)	0.218
Documented preadmission statin use (*n*, %)	21/276 (7.6)	3/13 (23.1)	5/39 (12.8)	13/224 (5.8)	0.031
Documented preadmission beta-blocker use (*n*, %)	9/276 (3.3)	3/13 (23.1)	1/39 (2.6)	5/224 (2.2)	< 0.001

Values are median (IQR) unless otherwise indicated. eGFR is expressed in mL/min/1.73 m^2^ and was recalculated with the 2021 CKD-EPI creatinine equation. Denominators vary because of missing values. Diabetic retinopathy stage was defined by the worst eligible eye.

### Descriptive retinal phenotypes

Across 565 eyes in the categorical UACR cohort, higher UACR categories showed lower median overall and arterial density, lower foveal density, and lower fractal dimensions, together with wider upper distributions of lesion counts and areas (**Table 3**). Microaneurysm, hemorrhage, and exudate variables are presented only as descriptive image characteristics and were not included among the eight prespecified inferential vascular outcomes. These eye-level summaries are unadjusted and do not account for fellow-eye correlation or clinical differences between categories.

**Table 3:** Descriptive, automatically quantified retinal phenotypes according to UACR category.

Retinal phenotype	Overall (565 eyes)	< 30 mg/g (394 eyes)	30–299 mg/g (132 eyes)	≥ 300 mg/g (39 eyes)
Vessel density	0.0930 (0.0821, 0.1004)	0.0944 (0.0852, 0.1012)	0.0908 (0.0788, 0.0980)	0.0817 (0.0682, 0.0920)
Arterial density	0.0351 (0.0294, 0.0394)	0.0361 (0.0309, 0.0398)	0.0333 (0.0278, 0.0380)	0.0295 (0.0243, 0.0324)
Venous density	0.0571 (0.0514, 0.0618)	0.0576 (0.0527, 0.0617)	0.0555 (0.0505, 0.0622)	0.0520 (0.0462, 0.0602)
Mean vessel density within 3-mm foveal region	0.0658 (0.0527, 0.0732)	0.0669 (0.0562, 0.0733)	0.0638 (0.0505, 0.0736)	0.0513 (0.0280, 0.0642)
Mean vessel density within 5-mm foveal region	0.0902 (0.0789, 0.0987)	0.0919 (0.0824, 0.0995)	0.0880 (0.0754, 0.0978)	0.0807 (0.0660, 0.0892)
Vascular fractal dimension	1.574 (1.550, 1.590)	1.578 (1.556, 1.591)	1.567 (1.542, 1.587)	1.541 (1.510, 1.571)
Arterial fractal dimension	1.394 (1.360, 1.413)	1.398 (1.370, 1.415)	1.383 (1.352, 1.408)	1.356 (1.324, 1.378)
Venous fractal dimension	1.468 (1.447, 1.483)	1.470 (1.451, 1.484)	1.462 (1.446, 1.483)	1.442 (1.420, 1.481)
No. of microaneurysms	1.0 (0.0, 2.0)	0.0 (0.0, 1.0)	1.0 (0.0, 3.0)	2.0 (0.5, 15.0)
No. of hemorrhages	0.0 (0.0, 0.0)	0.0 (0.0, 0.0)	0.0 (0.0, 1.0)	1.0 (0.0, 12.0)
Hemorrhage area (mm^2^)	0.000 (0.000, 0.000)	0.000 (0.000, 0.000)	0.000 (0.000, 0.008)	0.013 (0.000, 0.609)
No. of hard exudates	0.0 (0.0, 0.0)	0.0 (0.0, 0.0)	0.0 (0.0, 0.0)	0.0 (0.0, 0.5)
Hard exudate area (mm^2^)	0.000 (0.000, 0.000)	0.000 (0.000, 0.000)	0.000 (0.000, 0.000)	0.000 (0.000, 0.001)

Values are median (IQR). Nine patients with unambiguous inequality-reported UACR results were retained; one patient with < 30.46 mg/g was excluded because the reporting boundary crossed the category threshold. Microaneurysm, hemorrhage, and exudate variables are descriptive only and were not included among the eight prespecified inferential vascular outcomes. *p*-values were omitted because observations include correlated fellow eyes.

The 541-eye eGFR cohort showed a similar descriptive gradient, with lower vascular-density and fractal-dimension values in the lower-eGFR groups and greater lesion burden across several distributions (**Table 4**). Lesion-related variables were descriptive only and were not inferential outcomes. These patterns motivated, but do not substitute for, the clustered multivariable analyses.

**Table 4:** Descriptive, automatically quantified retinal phenotypes according to 2021 CKD-EPI eGFR category.

Retinal phenotype	Overall (541 eyes)	< 60 (24 eyes)	60–89 (77 eyes)	≥ 90 (440 eyes)
Vessel density	0.0931 (0.0826, 0.1008)	0.0809 (0.0580, 0.0939)	0.0852 (0.0742, 0.0961)	0.0943 (0.0852, 0.1019)
Arterial density	0.0351 (0.0297, 0.0394)	0.0284 (0.0186, 0.0329)	0.0312 (0.0256, 0.0376)	0.0359 (0.0312, 0.0397)
Venous density	0.0574 (0.0515, 0.0622)	0.0522 (0.0384, 0.0587)	0.0542 (0.0464, 0.0603)	0.0578 (0.0530, 0.0625)
Mean vessel density within 3-mm foveal region	0.0667 (0.0534, 0.0739)	0.0517 (0.0202, 0.0596)	0.0569 (0.0422, 0.0738)	0.0672 (0.0564, 0.0743)
Mean vessel density within 5-mm foveal region	0.0902 (0.0794, 0.0993)	0.0768 (0.0501, 0.0921)	0.0832 (0.0695, 0.0952)	0.0916 (0.0825, 0.0998)
Vascular fractal dimension	1.574 (1.551, 1.590)	1.539 (1.471, 1.576)	1.554 (1.524, 1.587)	1.577 (1.556, 1.592)
Arterial fractal dimension	1.394 (1.363, 1.413)	1.353 (1.265, 1.386)	1.367 (1.337, 1.408)	1.396 (1.371, 1.415)
Venous fractal dimension	1.469 (1.447, 1.484)	1.448 (1.385, 1.478)	1.457 (1.428, 1.481)	1.471 (1.452, 1.485)
No. of microaneurysms	1.0 (0.0, 2.0)	3.5 (0.0, 16.5)	1.0 (0.0, 2.0)	1.0 (0.0, 2.0)
No. of hemorrhages	0.0 (0.0, 0.0)	1.5 (0.0, 8.0)	0.0 (0.0, 1.0)	0.0 (0.0, 0.0)
Hemorrhage area (mm^2^)	0.000 (0.000, 0.000)	0.034 (0.000, 0.425)	0.000 (0.000, 0.012)	0.000 (0.000, 0.000)
No. of hard exudates	0.0 (0.0, 0.0)	0.0 (0.0, 1.2)	0.0 (0.0, 0.0)	0.0 (0.0, 0.0)
Hard exudate area (mm^2^)	0.000 (0.000, 0.000)	0.000 (0.000, 0.004)	0.000 (0.000, 0.000)	0.000 (0.000, 0.000)

Values are median (IQR). eGFR is expressed in mL/min/1.73 m^2^. Microaneurysm, hemorrhage, and exudate variables are descriptive only and were not included among the eight prespecified inferential vascular outcomes. *p*-values are omitted because observations include correlated fellow eyes.

### Primary separate models

The exact-UACR core model included 485 eyes from 248 patients (**Table 5**). Each doubling of UACR was nominally associated with lower arterial density (β = − 0.00054; 95% CI, − 0.00096 to − 0.00012; *p* = 0.012), but the association did not survive FDR correction (*q* = 0.097). No other UACR coefficient was significant after correction. Results were similarly null after medication expansion, eye-specific diabetic retinopathy adjustment, generalized estimating equations (GEE) analysis, and additional interval adjustment (**Supplementary Tables S4**–**S6**, **S10**).

**Table 5:** Primary separate multivariable associations of UACR and 2021 CKD-EPI eGFR with retinal vascular architecture.

Exposure	Retinal phenotype	Eyes	Patients	β (95% CI)	Standardized β	*p*-value	FDR *q*
Per doubling of UACR	Vessel density	485	248	− 0.00062 (− 0.00150, 0.00026)	− 0.074	0.166	0.266
Per doubling of UACR	Arterial density	485	248	− 0.00054 (− 0.00096, − 0.00012)	− 0.133	0.012	0.097
Per doubling of UACR	Venous density	485	248	− 0.00009 (− 0.00062, 0.00045)	− 0.017	0.753	0.860
Per doubling of UACR	Mean vessel density within 3-mm foveal region	485	248	− 0.00078 (− 0.00176, 0.00019)	− 0.085	0.115	0.266
Per doubling of UACR	Mean vessel density within 5-mm foveal region	485	248	− 0.00069 (− 0.00159, 0.00022)	− 0.074	0.136	0.266
Per doubling of UACR	Vascular fractal dimension	485	248	− 0.00130 (− 0.00401, 0.00140)	− 0.050	0.343	0.458
Per doubling of UACR	Arterial fractal dimension	485	248	− 0.00290 (− 0.00618, 0.00037)	− 0.083	0.082	0.266
Per doubling of UACR	Venous fractal dimension	485	248	− 0.00010 (− 0.00254, 0.00233)	− 0.004	0.934	0.934
Per 10 mL/min/1.73 m^2^ lower eGFR	Vessel density	509	260	− 0.00105 (− 0.00202, − 0.00008)	− 0.122	0.034	0.090
Per 10 mL/min/1.73 m^2^ lower eGFR	Arterial density	509	260	− 0.00053 (− 0.00099, − 0.00007)	− 0.129	0.025	0.090
Per 10 mL/min/1.73 m^2^ lower eGFR	Venous density	509	260	− 0.00052 (− 0.00110, 0.00006)	− 0.103	0.080	0.094
Per 10 mL/min/1.73 m^2^ lower eGFR	Mean vessel density within 3-mm foveal region	509	260	− 0.00136 (− 0.00247, − 0.00025)	− 0.144	0.017	0.090
Per 10 mL/min/1.73 m^2^ lower eGFR	Mean vessel density within 5-mm foveal region	509	260	− 0.00089 (− 0.00189, 0.00011)	− 0.095	0.082	0.094
Per 10 mL/min/1.73 m^2^ lower eGFR	Vascular fractal dimension	509	260	− 0.00272 (− 0.00554, 0.00011)	− 0.100	0.060	0.094
Per 10 mL/min/1.73 m^2^ lower eGFR	Arterial fractal dimension	509	260	− 0.00331 (− 0.00666, 0.00005)	− 0.104	0.054	0.094
Per 10 mL/min/1.73 m^2^ lower eGFR	Venous fractal dimension	509	260	− 0.00206 (− 0.00461, 0.00049)	− 0.086	0.113	0.113

Eye-level linear regression used patient-clustered robust standard errors and adjusted for age, gender, BMI, diabetes duration, HbA1c, and systolic blood pressure. Continuous UACR analyses included exact numeric values only. FDR correction was applied separately for UACR and eGFR across the eight *a priori* selected outcomes.

The CKD-EPI core model included 509 eyes from 260 patients (**Table 5**). Each 10 mL/min/1.73 m^2^ lower eGFR was nominally associated with lower overall vessel density (*p* = 0.034), arterial density (*p* = 0.025), and 3-mm foveal vessel density (*p* = 0.017), with borderline associations for several fractal measures. None remained significant after FDR correction (minimum *q* = 0.090). Medication expansion, diabetic retinopathy adjustment, GEE analysis, and interval adjustment produced similar effect directions but did not cross the corrected threshold (**Supplementary Tables S4**–**S6**, **S10**).

### Joint and exploratory sensitivity analyses

The joint core model included 431 eyes from 220 patients with exact UACR and CKD-EPI eGFR (**Table 6**). In exploratory same-cohort models restricted to this complete-case cohort, the UACR-only model showed no FDR-significant association, whereas the eGFR-only model was associated with all eight vascular outcomes after FDR correction (*q* = 0.029–0.045; **Supplementary Table S7A**). Thus, the stronger eGFR signal in the joint cohort was already present before UACR was added and may reflect complete-case cohort composition rather than statistical suppression by UACR. In the mutually adjusted model, UACR remained null (all *q* ≥ 0.746), and seven eGFR associations remained significant (all *q* = 0.037); the 5-mm foveal-density coefficient was attenuated below the corrected threshold (*q* = 0.064). The medication-expanded joint model gave the same seven-outcome pattern (**Supplementary Table S7B**). A direct patient-level comparison of the joint complete-case cohort with the 117 final-cohort patients not included in that analysis showed fewer women among complete cases (27.3% vs. 37.6%; standardized mean difference (SMD) = − 0.222), a higher prevalence of diabetic retinopathy (37.7% vs. 29.9%; SMD = 0.166), and a higher prevalence of documented smoking history (41.8% vs. 29.1%; SMD = 0.269). BMI showed a smaller imbalance (SMD = 0.108), whereas differences in age, diabetes duration, systolic blood pressure, and HbA1c were small (**Supplementary Table S12E**). These findings support the possibility of nonrandom complete-case selection.

**Table 6:** Joint core model including both UACR and 2021 CKD-EPI eGFR.

Exposure	Retinal phenotype	Eyes	Patients	β (95% CI)	Standardized β	*p*-value	FDR *q*
Per doubling of UACR	Vessel density	431	220	− 0.00022 (− 0.00112, 0.00068)	− 0.027	0.635	0.746
Per doubling of UACR	Arterial density	431	220	− 0.00034 (− 0.00077, 0.00009)	− 0.088	0.120	0.746
Per doubling of UACR	Venous density	431	220	0.00012 (− 0.00042, 0.00067)	0.026	0.653	0.746
Per doubling of UACR	Mean vessel density within 3− mm foveal region	431	220	− 0.00027 (− 0.00127, 0.00073)	− 0.030	0.598	0.746
Per doubling of UACR	Mean vessel density within 5− mm foveal region	431	220	− 0.00027 (− 0.00121, 0.00067)	− 0.030	0.573	0.746
Per doubling of UACR	Vascular fractal dimension	431	220	− 0.00025 (− 0.00300, 0.00250)	− 0.010	0.858	0.858
Per doubling of UACR	Arterial fractal dimension	431	220	− 0.00182 (− 0.00509, 0.00144)	− 0.064	0.273	0.746
Per doubling of UACR	Venous fractal dimension	431	220	0.00081 (− 0.00169, 0.00331)	0.036	0.522	0.746
Per 10 mL/min/1.73 m^2^ lower eGFR	Vessel density	431	220	− 0.00128 (− 0.00234, − 0.00021)	− 0.153	0.019	0.037
Per 10 mL/min/1.73 m^2^ lower eGFR	Arterial density	431	220	− 0.00055 (− 0.00105, − 0.00005)	− 0.136	0.033	0.037
Per 10 mL/min/1.73 m^2^ lower eGFR	Venous density	431	220	− 0.00073 (− 0.00136, − 0.00010)	− 0.149	0.024	0.037
Per 10 mL/min/1.73 m^2^ lower eGFR	Mean vessel density within 3-mm foveal region	431	220	− 0.00171 (− 0.00298, − 0.00044)	− 0.187	0.008	0.037
Per 10 mL/min/1.73 m^2^ lower eGFR	Mean vessel density within 5-mm foveal region	431	220	− 0.00107 (− 0.00219, 0.00006)	− 0.117	0.064	0.064
Per 10 mL/min/1.73 m^2^ lower eGFR	Vascular fractal dimension	431	220	− 0.00358 (− 0.00659, − 0.00057)	− 0.140	0.020	0.037
Per 10 mL/min/1.73 m^2^ lower eGFR	Arterial fractal dimension	431	220	− 0.00393 (− 0.00742, − 0.00044)	− 0.134	0.028	0.037
Per 10 mL/min/1.73 m^2^ lower eGFR	Venous fractal dimension	431	220	− 0.00303 (− 0.00576, − 0.00030)	− 0.131	0.030	0.037

Exact numeric log2-transformed UACR and eGFR, scaled per 10 mL/min/1.73 m^2^ lower value, were entered simultaneously. Models were adjusted for age, gender, BMI, diabetes duration, HbA1c, and systolic blood pressure. FDR correction was performed separately for the eight UACR coefficients and the eight eGFR coefficients. The complete-case sample comprised 431 eyes from 220 patients.

Given that smoking history was complete in all 337 final-cohort patients and differed between the joint complete-case and excluded groups, *post hoc* smoking-adjusted sensitivity analyses were performed (**Supplementary Table S14**). In the 485-eye/248-patient UACR core cohort, additional smoking adjustment strengthened the arterial-density association (β = − 0.00059 per UACR doubling; 95% CI, − 0.00101 to − 0.00017; *p* = 0.006; *q* = 0.048), whereas the other seven UACR outcomes remained nonsignificant after FDR correction. In the same 431-eye/220-patient joint cohort, an eGFR-only model additionally adjusted for smoking retained FDR significance for overall vessel density, arterial density, 3-mm foveal vessel density, overall fractal dimension, and arterial fractal dimension (all *q* = 0.044), but not for venous density, 5-mm foveal vessel density, or venous fractal dimension (*q* = 0.062–0.071). When UACR, eGFR, and smoking were entered together, none of the eight eGFR coefficients remained FDR-significant (*q* = 0.074–0.104), and UACR remained nonsignificant. These *post hoc* results further support cautious interpretation of the complete-case signal.

Exploratory alternative eGFR analyses yielded stronger eGFR-related associations. Using the laboratory-reported Chinese-modified MDRD estimate, all eight outcomes were significant after FDR correction. When CKD-EPI eGFR was restricted to values below 120 mL/min/1.73 m^2^, overall and arterial density, both foveal density measures, overall fractal dimension, and arterial fractal dimension remained significant, whereas venous density and venous fractal dimension did not (**Supplementary Tables S8**, **S9**). As these analyses were secondary and FDR was controlled separately within each analytical family, they are interpreted as hypothesis-generating.

In prespecified secondary categorical analyses, eGFR < 90 mL/min/1.73 m^2^ was associated with lower overall, arterial, and venous density; both foveal density measures; overall fractal dimension; and arterial fractal dimension relative to eGFR 90–119 mL/min/1.73 m^2^. The ≥ 120-mL/min/1.73 m^2^ category was not associated with an adverse vascular profile after FDR correction (**Supplementary Table S10**). As prespecified in the Methods, the 120-mL/min/1.73 m^2^ boundary defined an analytical high-eGFR stratum rather than a diagnostic definition of hyperfiltration. Given that eGFR-based definitions of hyperfiltration vary with age, gender, population, and estimating method, this category should be interpreted as a high-eGFR stratum rather than confirmed hyperfiltration in individual participants. Restricted cubic splines identified FDR-significant nonlinearity only for 5-mm foveal vessel density (*q* = 0.039); several other outcomes were nominally nonlinear but did not survive correction (**Supplementary Table S11**).

Exploratory analyses restricted to a laboratory-report-to-image interval of ≤ 3 days retained similar directions and nominal signals, but no association survived FDR correction. Analyses restricted to identical laboratory-report and image dates were based on approximately 109–110 patients and were null, with substantially wider confidence intervals (**Supplementary Table S10**).

## Discussion

The principal finding is that neither UACR nor 2021 CKD-EPI eGFR was associated with any of the eight retinal vascular outcomes after FDR correction in the primary separate core models. UACR showed only a nominal association with arterial density in the primary analysis; an isolated arterial-density association crossed the FDR threshold only in a *post hoc* smoking-adjusted model. Lower eGFR showed concordant but statistically uncertain directions in the primary model; the broader FDR-significant pattern emerged only in exploratory complete-case, restricted-range, and alternative-equation analyses and was further attenuated by *post hoc* smoking adjustment. These secondary results are therefore hypothesis-generating and should not be assigned the same evidential weight as the FDR-negative primary analyses, particularly because multiplicity was controlled within model families rather than across the full set of analytical choices.

The weak UACR results are biologically plausible and methodologically informative. Albumin excretion is a clinically important marker of glomerular and endothelial injury, but a single inpatient UACR can vary with blood pressure, glycemic exposure, physical activity, inflammation, hydration, and collection conditions. Ten patients had results reported solely as record-specific inequalities. Excluding those values from continuous analyses avoided unverifiable substitutions, but also removed observations from the tails of the exposure distribution. The primary arterial-density association did not survive multiplicity correction or persist in the joint, retinopathy-adjusted, or GEE models. In a *post hoc* model additionally adjusted for smoking history, however, the arterial-density association became FDR-significant (β = − 0.00059 per UACR doubling; 95% CI, − 0.00101 to − 0.00017; *p* = 0.006; *q* = 0.048), while the other seven UACR outcomes remained nonsignificant after correction. Given that smoking adjustment was not prespecified and this was an isolated result across many analytical specifications, it should be regarded as hypothesis-generating rather than as evidence of a stable independent UACR effect.

The eGFR findings require similarly careful interpretation. In the broader eGFR core sample, several coefficients had *p*-values below 0.05 but *q*-values near 0.09. When the eGFR-only model was refitted in the same 431-eye, 220-patient joint complete-case cohort, all eight outcomes were already FDR-significant; adding UACR modestly attenuated the coefficients and left seven outcomes significant. This argues against statistical suppression by UACR, but it does not establish a stronger biological relation in the smaller cohort. The direct 220-versus-117 comparison showed that the joint complete-case cohort differed particularly in gender, diabetic retinopathy burden, and smoking history. In *post hoc* smoking-adjusted analyses restricted to the same joint cohort, the eGFR-only model retained FDR significance for overall vessel density, arterial density, 3-mm foveal vessel density, overall fractal dimension, and arterial fractal dimension (all *q* = 0.044), but not for venous density, 5-mm foveal vessel density, or venous fractal dimension (*q* = 0.062–0.071). After simultaneous adjustment for UACR and smoking, none of the eight eGFR coefficients survived FDR correction (*q* = 0.074–0.104), and UACR also remained nonsignificant. Together, these findings suggest that complete-case composition and residual vascular confounding may contribute to the stronger eGFR pattern. We therefore regard the complete-case findings as exploratory and in need of external replication. Multiple imputation was not used for missing kidney-marker exposures because doing so would require assumptions that are strong and unverifiable about the mechanism governing clinical testing and missing exposure values.

The restricted-range and categorical analyses were prespecified secondary analyses intended to examine model dependence across the upper eGFR range rather than to establish a clinical breakpoint. The eGFR distribution was concentrated at relatively high values, and the ≥ 120 mL/min/1.73 m^2^ category did not show a consistently adverse retinal profile. Fixed GFR/eGFR thresholds around 120 mL/min/1.73 m^2^ have precedent in type 2 diabetes hyperfiltration studies: Ruggenenti et al. used measured GFR ≥ 120 mL/min/1.73 m^2^, and Tricò et al. used eGFR > 120 mL/min/1.73 m^2^ (38, 39). Oh et al. separately analyzed eGFR > 120 and 90–119 mL/min/1.73 m^2^, while defining hyperfiltration formally by an age- and gender-specific percentile (40). Taken together, these studies support the prespecified analytical stratification but also underscore that hyperfiltration does not have a single universally accepted fixed eGFR definition. Creatinine-based equations are also less precise at high filtration levels and may be influenced by muscle mass and nutritional status. Restriction to eGFR < 120 and the categorical contrast of < 90 versus 90–119 yielded clearer associations, whereas the spline analysis identified FDR-significant nonlinearity only for the 5-mm foveal measure. These secondary findings therefore remain supportive and hypothesis-generating; they do not establish measured glomerular hyperfiltration, a biological breakpoint at 120 mL/min/1.73 m^2^, or a subgroup in which retinal measurements have demonstrated clinical utility.

Vessel density and fractal dimension describe related but distinct properties of the retinal vascular image. Density reflects the proportion of a color fundus photograph occupied by detectable segmented vessels, whereas fractal dimension summarizes branching complexity (11, 32, 33). These measures are not equivalent to OCTA-derived capillary vessel density or perfusion and are not direct measures of histological vessel loss. Segmentation performance, artery-vein classification, foveal localization, analysis masks, image quality, camera positioning, and patient-specific ocular magnification can affect the measurements. As test–retest technical variability of the exact EVisionAI 4.0 configuration was not measured in this cohort, the biological importance of small absolute differences cannot be judged against a directly observed repeatability threshold. The modest standardized coefficients therefore support, at most, group-level phenotyping and mechanistic hypothesis generation rather than individual diagnosis or risk prediction.

Previous studies have reported both positive and null associations between retinal vascular measures and kidney measures (13–19). Recent deep-learning work has extended the field toward disease classification: DeepDKD was developed to detect diabetic kidney disease and distinguish isolated diabetic nephropathy from nondiabetic kidney disease, while KIDS was developed for CKD screening and common pathological-type identification from retinal images (34, 35). Those studies address prediction or pathology classification rather than interpretable vascular correlates of contemporaneous UACR and eGFR. In the present study, heterogeneity across analyses may reflect the eGFR equation, the upper eGFR range, mutual adjustment for albuminuria, temporal eligibility, multiplicity correction, complete-case selection, and handling of two-eye data. The laboratory-reported Chinese-modified MDRD equation produced substantially higher estimates than 2021 CKD-EPI, although the two were strongly correlated; stronger associations with the MDRD estimate do not establish superior biological validity and may partly reflect scaling and distributional differences.

Temporal matching was a central design feature. The median absolute laboratory-report-to-image interval was 1 day, and almost 98% of eye records were within 3 days. Results were little changed after direct adjustment for the interval or restriction to ≤ 3 days, but analyses restricted to identical laboratory-report and image dates were null and imprecise because the effective sample was reduced by more than half. This pattern cannot determine whether short-term biological variation or loss of power is the dominant explanation. It does, however, show why the full ≤ 7-day analysis and tighter-window analyses should be reported together rather than claiming that all kidney and retinal measurements were simultaneous.

This study has several strengths. The source data were audited at the eye and patient levels, temporal eligibility was applied using a directly recorded laboratory-report-to-image interval, and all UACR-related analyses were rerun after excluding nonnumeric reports from continuous models. The creatinine assay and its IDMS traceability were documented; eGFR was recalculated with a contemporary race-free equation and compared with the laboratory-reported formula. Bilateral-eye correlation was addressed with patient-clustered variance and GEE. The primary covariate set was separated from medication-expanded and diabetic-retinopathy-adjusted models, and multiplicity correction was defined within *a priori*-selected outcome families.

The limitations are substantial. The retrospective cross-sectional design precludes temporal or causal inference. The cohort came from a single inpatient endocrinology service, had high HbA1c values, and included only 13 patients with eGFR < 60 mL/min/1.73 m^2^; generalizability to community populations or patients with established advanced kidney dysfunction is therefore limited. Kidney markers were measured during one hospitalization, so the study cannot establish persistent CKD, diabetic kidney disease, or biopsy-defined diabetic nephropathy. Repeated UACR, cystatin C, measured GFR, and formal adjudication of chronic kidney disease or acute kidney injury were unavailable, and UACR collection conditions could not be standardized. Excluding patients without a verifiable laboratory-report-to-image interval and using complete-case kidney-marker analyses may have introduced selection bias, as supported by the direct joint complete-case comparison in **Supplementary Table S12E**. Temporal matching used laboratory report date rather than specimen collection time. Medication variables reflected documented history rather than pharmacy-verified exposure. Smoking history was complete and was examined in a *post hoc* sensitivity analysis, but lipid measurements were incomplete, and a structured cardiovascular-comorbidity variable was unavailable; residual systemic vascular confounding therefore remains possible. Diabetic retinopathy was graded by one masked ophthalmologist, so intergrader agreement was not available. When more than one quality-eligible photograph was available for an eye, the platform retained the image with the highest automatically assessed diabetic retinopathy severity. As this rule is pathology-dependent, it could theoretically enrich abnormal retinal phenotypes; however, platform audit data showed that only eight of 872 eyes (0.9%) were subject to this rule. The alternative quality-eligible candidate photographs and their complete quantitative outputs could not be retrieved retrospectively from the archived platform records; therefore, a neutral image-selection sensitivity analysis using the first, highest-quality, or randomly selected eligible photograph was not technically feasible. Residual image-selection bias therefore cannot be completely excluded. Image-specific pixel-pitch calibration corrects image/camera scale but not individual ocular magnification; axial length and refractive error were unavailable. All quantitative vascular outputs were generated automatically without manual correction, reducing investigator-dependent modification of the image-analysis results. Nevertheless, published information on EVisionAI-related segmentation supports the general platform approach but does not provide version-specific validation for every EVisionAI 4.0 component. In particular, artery–vein classification accuracy, quantitative foveal-localization error, vessel-density repeatability, fractal-dimension repeatability, and performance across diabetic retinopathy severity were not independently validated against manual or repeated-acquisition reference measurements in this cohort.

Prospective multicenter studies should use standardized urine collection, repeated kidney measurements, cystatin C or measured filtration where feasible, protocol-defined neutral image-selection rules, repeated retinal acquisition, and independent validation of quantitative vascular outputs. Longitudinal studies would be required before evaluating whether retinal architecture predicts eGFR decline or albuminuria progression or adds clinically useful information beyond established kidney-risk factors.

## Conclusion

Among hospitalized adults with type 2 diabetes and a laboratory-report-to-image interval no greater than 7 days, neither UACR nor 2021 CKD-EPI eGFR was associated with the eight automatically quantified retinal vascular phenotypes after FDR correction in the primary separate models. Exploratory complete-case and sensitivity analyses suggested a broader, model-dependent eGFR-related pattern, whereas UACR remained largely null.

These cross-sectional findings are hypothesis-generating. They do not establish persistent CKD or diabetic kidney disease, do not demonstrate a clinically meaningful eGFR breakpoint, and do not establish diagnostic, predictive, or incremental clinical utility of retinal vascular measurements. Independent prospective and longitudinal validation is required before any clinical application is considered.

## Data Availability

The datasets used and/or analyzed during the current study are available from the corresponding authors upon reasonable request and subject to ethical approval.

## References

[1] American Diabetes Association Professional Practice Committee for Diabetes . 11. Chronic kidney disease and risk management: Standards of care in diabetes—2026. Diabetes Care. (2026) 49:S246–60. doi: 10.2337/dc26-S011 41358881 PMC12690176

[2] Kidney Disease: Improving Global Outcomes (KDIGO) CKD Work Group . KDIGO 2024 clinical practice guideline for the evaluation and management of chronic kidney disease. Kidney Int. (2024) 105:S117–314. doi: 10.1016/j.kint.2023.10.018 38490803

[3] Kidney Disease: Improving Global Outcomes (KDIGO) Diabetes Work Group . KDIGO 2022 clinical practice guideline for diabetes management in chronic kidney disease. Kidney Int. (2022) 102:S1–S127. doi: 10.1016/j.kint.2022.06.008 36272764

[4] American Diabetes Association Professional Practice Committee for Diabetes . 12. Retinopathy, neuropathy, and foot care: Standards of care in diabetes—2026. Diabetes Care. (2026) 49:S261–76. doi: 10.2337/dc26-S012 41358886 PMC12690177

[5] Solomon SD Chew E Duh EJ Sobrin L Sun JK VanderBeek BL . Diabetic retinopathy: a position statement by the American Diabetes Association. Diabetes Care. (2017) 40:412–8. doi: 10.2337/dc16-2641 28223445 PMC5402875

[6] Yau JW Rogers SL Kawasaki R Lamoureux EL Kowalski JW Bek T . Global prevalence and major risk factors of diabetic retinopathy. Diabetes Care. (2012) 35:556–64. doi: 10.2337/dc11-1909 22301125 PMC3322721

[7] Wilkinson CP Ferris FL Klein RE Lee PP Agardh CD Davis M . Proposed international clinical diabetic retinopathy and diabetic macular edema disease severity scales. Ophthalmology. (2003) 110:1677–82. doi: 10.1016/S0161-6420(03)00475-5 13129861

[8] Cheung N Mitchell P Wong TY . Diabetic retinopathy. Lancet. (2010) 376:124–36. doi: 10.1016/S0140-6736(09)62124-3 20580421

[9] Penno G Solini A Orsi E Bonora E Fondelli C Trevisan R . Non-albuminuric renal impairment is a strong predictor of mortality in individuals with type 2 diabetes: the Renal Insufficiency And Cardiovascular Events (RIACE) Italian multicentre study. Diabetologia. (2018) 61:2277–89. doi: 10.1007/s00125-018-4691-2 30032426

[10] Yamamoto Y Hanai K Mori T Yokoyama Y Yoshida N Murata H . Kidney outcomes and all-cause mortality in people with type 2 diabetes exhibiting non-albuminuric kidney insufficiency. Diabetologia. (2022) 65:234–45. doi: 10.1007/s00125-021-05590-5 34739552

[11] Gao Y Xu L He N Ding Y Zhao W Meng T . A narrative review of retinal vascular parameters and the applications (Part I): measuring methods. Brain Circ. (2023) 9:121–8. doi: 10.4103/bc.bc_8_23 38020955 PMC10679626

[12] Yang J Liu Z . Mechanistic pathogenesis of endothelial dysfunction in diabetic nephropathy and retinopathy. Front Endocrinol (Lausanne). (2022) 13:816400. doi: 10.3389/fendo.2022.816400 35692405 PMC9174994

[13] Aronov M Allon R Stave D Belkin M Margalit E Fabian ID . Retinal vascular signs as screening and prognostic factors for chronic kidney disease: a systematic review and meta-analysis of current evidence. J Pers Med. (2021) 11:665. doi: 10.3390/jpm11070665 34357132 PMC8307097

[14] Paterson EN Cardwell C MacGillivray TJ Trucco E Doney AS Foster P . Investigation of associations between retinal microvascular parameters and albuminuria in UK Biobank: a cross-sectional case-control study. BMC Nephrol. (2021) 22:72. doi: 10.1186/s12882-021-02273-6 33632154 PMC7908698

[15] Yip W Ong PG Teo BW Cheung CY Tai ES Cheng CY . Retinal vascular imaging markers and incident chronic kidney disease: a prospective cohort study. Sci Rep. (2017) 7:9374. doi: 10.1038/s41598-017-09204-2 28839244 PMC5570935

[16] Nusinovici S Sabanayagam C Lee KE Zhang L Cheung CY Tai ES . Retinal microvascular signs and risk of diabetic kidney disease in Asian and White populations. Sci Rep. (2021) 11:4898. doi: 10.1038/s41598-021-84464-7 33649427 PMC7921402

[17] Kim JS Kim ES Hwang HS Jeong KH Yu SY Kim K . Association between albuminuria and retinal microvascular parameters measured with swept-source optical coherence tomography angiography in patients with diabetic retinopathy. PLoS One. (2024) 19:e0295768. doi: 10.1371/journal.pone.0295768 38446750 PMC10917288

[18] Zhao X Liu Y Zhang W Meng L Lv B Lv C . Relationships between retinal vascular characteristics and renal function in patients with type 2 diabetes mellitus. Transl Vis Sci Technol. (2021) 10:20. doi: 10.1167/tvst.10.2.20 34003905 PMC7884293

[19] McKay GJ Paterson EN Maxwell AP Cardwell CC Wang R Hogg S . Retinal microvascular parameters are not associated with reduced renal function in a study of individuals with type 2 diabetes. Sci Rep. (2018) 8:3931. doi: 10.1038/s41598-018-22360-3 29500396 PMC5834527

[20] Xu X Gao B Ding W Wang Q Zhang M Tan T . Retinal image measurements and their association with chronic kidney disease in Chinese patients with type 2 diabetes: the NCD study. Acta Diabetol. (2021) 58:363–70. doi: 10.1007/s00592-020-01621-6 33098472

[21] Wang W He M Gong X Wang L Meng J Li Y . Association of renal function with retinal vessel density in patients with type 2 diabetes by using swept-source optical coherence tomographic angiography. Br J Ophthalmol. (2020) 104:1768–73. doi: 10.1136/bjophthalmol-2019-315450 32098859

[22] Ma R Zheng C Niu Y Cao D Hu Y Jin L . Changes of retinal microvascular parameters in patients with type 2 diabetes mellitus with or without diabetic kidney disease. Front Endocrinol (Lausanne). (2026) 17:1727260. doi: 10.3389/fendo.2026.1727260 41821728 PMC12975603

[23] Sabanayagam C Xu D Ting DSW Nusinovici S Banu R Hamzah H . A deep learning algorithm to detect chronic kidney disease from retinal photographs in community-based populations. Lancet Digit Health. (2020) 2:e295–302. doi: 10.1016/S2589-7500(20)30063-7 33328123

[24] Zhang K Liu X Xu J Yuan J Cai W Chen T . Deep-learning models for the detection and incidence prediction of chronic kidney disease and type 2 diabetes from retinal fundus images. Nat BioMed Eng. (2021) 5:533–45. doi: 10.1038/s41551-021-00745-6 34131321

[25] Zhao X Gu X Meng L Chen Y Zhao Q Cheng S . Screening chronic kidney disease through deep learning utilizing ultra-wide-field fundus images. NPJ Digit Med. (2024) 7:275. doi: 10.1038/s41746-024-01271-w 39375513 PMC11458603

[26] Joo YS Rim TH Koh HB Yi J Kim H Lee G . Non-invasive chronic kidney disease risk stratification tool derived from retina-based deep learning and clinical factors. NPJ Digit Med. (2023) 6:114. doi: 10.1038/s41746-023-00860-5 37330576 PMC10276847

[27] Inker LA Eneanya ND Coresh J Tighiouart H Wang D Sang Y . New creatinine- and cystatin C-based equations to estimate GFR without race. N Engl J Med. (2021) 385:1737–49. doi: 10.1056/NEJMoa2102953 34554658 PMC8822996

[28] Ma YC Zuo L Chen JH Luo Q Yu XQ Li Y . Modified glomerular filtration rate estimating equation for Chinese patients with chronic kidney disease. J Am Soc Nephrol. (2006) 17:2937–44. doi: 10.1681/ASN.2006040368 16988059

[29] Long T Xu Y Zou H Lu L Yuan T Dong Z . A generic pixel pitch calibration method for fundus camera via automated ROI extraction. Sensors (Basel). (2022) 22:8565. doi: 10.3390/s22218565 36366262 PMC9653591

[30] Benjamini Y Hochberg Y . Controlling the false discovery rate: a practical and powerful approach to multiple testing. J R Stat Soc Ser B Stat Methodol. (1995) 57:289–300. doi: 10.1111/j.2517-6161.1995.tb02031.x40046247

[31] von Elm E Altman DG Egger M Pocock SJ Gøtzsche PC Vandenbroucke JP . The Strengthening the Reporting of Observational Studies in Epidemiology (STROBE) statement: guidelines for reporting observational studies. J Clin Epidemiol. (2008) 61:344–9. doi: 10.1016/j.jclinepi.2007.11.008 18313558

[32] Fu Y Yusufu M Wang Y He M Shi D Wang R . Association of retinal microvascular density and complexity with incident coronary heart disease. Atherosclerosis. (2023) 380:117196. doi: 10.1016/j.atherosclerosis.2023.117196 37562159

[33] Lemmens S Devulder A Van Keer K Bierkens J De Boever P Stalmans I . Systematic review on fractal dimension of the retinal vasculature in neurodegeneration and stroke: assessment of a potential biomarker. Front Neurosci. (2020) 14:16. doi: 10.3389/fnins.2020.00016 32116491 PMC7025576

[34] Meng Z Guan Z Yu S Wu Y Zhao Y Shen J . Non-invasive biopsy diagnosis of diabetic kidney disease via deep learning applied to retinal images: a population-based study. Lancet Digit Health. (2025) 7:100868. doi: 10.1016/j.landig.2025.02.008 40312169

[35] Wu Q Li J Zhao L Liu D Wen J Wang Y . A noninvasive model for chronic kidney disease screening and common pathological type identification from retinal images. Nat Commun. (2025) 16:6962. doi: 10.1038/s41467-025-62273-0 40730556 PMC12307705

[36] Shi X Dong L Shao L Ling S Dong Z Niu Y . Measurement and characterization of retinal vascular morphology parameters based on artificial intelligence automated analysis technology. Chin J Exp Ophthalmol. (2024) 42:38–46. doi: 10.3760/cma.j.cn115989-20220715-0032630704229

[37] Li Y Zhang C Wang X Li S Cao D Liu C . Study on fundus characteristics of preschool children based on artificial intelligence quantitative technology. BMC Ophthalmol. (2026) 26:275. doi: 10.1186/s12886-026-04705-z 41882586 PMC13188452

[38] Ruggenenti P Porrini EL Gaspari F Motterlini N Cannata A Carrara F . Glomerular hyperfiltration and renal disease progression in type 2 diabetes. Diabetes Care. (2012) 35:2061–8. doi: 10.2337/dc11-2189 22773704 PMC3447826

[39] Tricò D Fadini GP Morieri ML Candido R Disoteo OE Frontoni S . Prevalence and risk factors of glomerular hyperfiltration in adults with type 2 diabetes: a population-based study. Diabetes Metab Res Rev. (2024) 40:e3782. doi: 10.1002/dmrr.3782 38402454

[40] Oh SW Han KH Han SY . Associations between renal hyperfiltration and serum alkaline phosphatase. PLoS One. (2015) 10:e0122921. doi: 10.1371/journal.pone.0122921 25853240 PMC4390244

