# Supplementary material for: Albuminuria, kidney function, and automatically quantified retinal vascular phenotypes in hospitalized adults with type 2 diabetes
Source: Front Endocrinol (Lausanne). 2026 Sep 28;17:1957415. doi: 10.3389/fendo.2026.1957415 (PMC13654698; doi:10.3389/fendo.2026.1957415)
Supplement: Supplementary file 1 [file Supplementaryfile1.docx]

# **Supplementary Material**

# **Contents**

Supplementary Table S1. Source-data audit, cohort screening, and temporal matching

Supplementary Table S2. Laboratory assays, calculation formulas, and reporting rules

Supplementary Table S3. Patient-level comparison of eGFR equations

Supplementary Table S4. Medication-expanded models

Supplementary Table S5. Diabetic-retinopathy-adjusted models

Supplementary Table S6. Generalized estimating equation analyses

Supplementary Table S7A. Single-exposure and mutually adjusted models in the same joint complete-case cohort

Supplementary Table S7B. Medication-expanded joint model

Supplementary Table S8. Sensitivity analysis using laboratory-reported Chinese-modified MDRD eGFR

Supplementary Table S9. Sensitivity analysis restricted to 2021 CKD-EPI eGFR <120 mL/min/1.73 m²

Supplementary Table S10. eGFR category, temporal-window, and interval-adjusted analyses

Supplementary Table S11. Restricted cubic spline tests for nonlinearity

Supplementary Table S12. Kidney-marker availability and complete-case comparisons

Supplementary Table S12E. Joint core complete-case cohort versus excluded patients

Supplementary Table S13. Operational definitions of automatically quantified retinal phenotypes

Supplementary Table S14. Post hoc smoking-adjusted sensitivity analyses

Supplementary Figure S1. Box-counting calculation of retinal vascular fractal dimension

**Supplementary Table S1. Source-data audit, cohort screening, and temporal matching.**

| **Cohort definition** | **No. of eyes** | **No. of patients** |
| --- | --- | --- |
| Workbook rows | 1,060 | - |
| Populated source eye-level records | 1,058 | 529 |
| After prior clinical, ocular, and image-quality exclusions | 872 | 450 |
| Age 18-80 years | 861 | 444 |
| Laboratory-image interval available | 750 | 387 |
| Absolute laboratory-image interval ≤7 days | 660 | 339 |
| Final cohort with complete eight core AI outputs | 658 | 337 |
| Bilateral-eye contributors | 642 eyes | 321 |
| Unilateral-eye contributors | 16 eyes | 16 |
| Any UACR record | 567 | 291 |
| Exact numeric UACR | 548 | 281 |
| Categorical UACR cohort | 565 | 290 |
| 2021 CKD-EPI eGFR available | 541 | 276 |
| Both exact UACR and eGFR available | 455 | 232 |
| UACR core complete case | 485 | 248 |
| eGFR core complete case | 509 | 260 |
| Joint core complete case | 431 | 220 |

Note. The source worksheet contained two header rows. The final cohort had a median absolute laboratory-image interval of 1 day (IQR, 0-1); 42.6% were same-day, 81.2% were within 1 day, 97.9% were within 3 days, and the maximum interval was 6 days. Missing interval values could not satisfy temporal eligibility and were excluded. A retrospective audit of the EVisionAI platform logs showed that 8 of the 872 eyes (0.9%) remaining after the initial clinical, ocular, and image-quality exclusions had two or more quality-eligible candidate photographs and were subject to the predefined multiple-image selection rule.

**Supplementary Table S2. Laboratory assays, calculation formulas, and reporting rules.**

| **Analyte** | **Method** | **Analyzer** | **Reagent** | **Reportable range / traceability** |
| --- | --- | --- | --- | --- |
| Urinary microalbumin | Immunoturbidimetry | Beckman Coulter IMMAGE 800 | Beckman urinary microalbumin reagent | 0-1800 mg/dL |
| Urinary creatinine | Sarcosine oxidase method | Beckman Coulter AU5800 | Beckman urinary creatinine reagent | 0-50,000 μmol/L |
| Serum creatinine | Creatininase-creatinase-sarcosine oxidase enzymatic method | Beckman Coulter AU5800 | Creatinine (Enzymatic) OSR61204 | IDMS-traceable through NIST SRM 967 |

UACR (mg/g) = MA × 10,000 / UCRE / 0.113, where MA is urinary microalbumin (mg/dL) and UCRE is urinary creatinine (μmol/L). The analyzers, reagents, measurement methods, formula, and reporting rules remained unchanged throughout the study period. When urinary albumin was outside the reportable range, the laboratory information system reported UACR as a record-specific inequality (<x or >x).

**Supplementary Table S3. Patient-level comparison of laboratory-reported Chinese-modified MDRD eGFR and 2021 CKD-EPI eGFR.**

| **Measure** | **Value** |
| --- | --- |
| Unique paired patients | 224 |
| 2021 CKD-EPI eGFR, median (IQR) | 103.7 (93.2, 112.3) |
| Laboratory-reported MDRD eGFR, median (IQR) | 132.7 (108.7, 160.9) |
| Paired difference (MDRD minus CKD-EPI), median (IQR) | 29.5 (14.2, 50.1) |
| Spearman correlation | ρ=0.875; P<0.001 |

**Note.** Each patient was counted once. eGFR values are in mL/min/1.73 m². Differences reflect equation structure and do not represent measured GFR error.

**Supplementary Table S4. Medication-expanded associations of UACR and 2021 CKD-EPI eGFR.**

| **Exposure** | **Retinal phenotype** | **Eyes** | **Patients** | **β (95% CI)** | **Standardized β** | **P value** | **FDR q** |
| --- | --- | --- | --- | --- | --- | --- | --- |
| Per doubling of UACR | Vessel density | 485 | 248 | -0.00057 (-0.00148, 0.00034) | -0.068 | 0.215 | 0.344 |
| Per doubling of UACR | Arterial density | 485 | 248 | -0.00051 (-0.00094, -0.00009) | -0.127 | 0.018 | 0.146 |
| Per doubling of UACR | Venous density | 485 | 248 | -0.00006 (-0.00061, 0.00048) | -0.013 | 0.822 | 0.939 |
| Per doubling of UACR | Mean vessel density within 3-mm foveal region | 485 | 248 | -0.00072 (-0.00172, 0.00028) | -0.078 | 0.159 | 0.325 |
| Per doubling of UACR | Mean vessel density within 5-mm foveal region | 485 | 248 | -0.00065 (-0.00157, 0.00026) | -0.071 | 0.162 | 0.325 |
| Per doubling of UACR | Vascular fractal dimension | 485 | 248 | -0.00111 (-0.00392, 0.00171) | -0.042 | 0.438 | 0.584 |
| Per doubling of UACR | Arterial fractal dimension | 485 | 248 | -0.00255 (-0.00600, 0.00090) | -0.073 | 0.147 | 0.325 |
| Per doubling of UACR | Venous fractal dimension | 485 | 248 | 0.00004 (-0.00247, 0.00256) | 0.002 | 0.972 | 0.972 |
| Per 10 mL/min/1.73 m² lower eGFR | Vessel density | 509 | 260 | -0.00106 (-0.00205, -0.00007) | -0.123 | 0.036 | 0.095 |
| Per 10 mL/min/1.73 m² lower eGFR | Arterial density | 509 | 260 | -0.00053 (-0.00100, -0.00006) | -0.130 | 0.026 | 0.095 |
| Per 10 mL/min/1.73 m² lower eGFR | Venous density | 509 | 260 | -0.00053 (-0.00112, 0.00007) | -0.105 | 0.081 | 0.095 |
| Per 10 mL/min/1.73 m² lower eGFR | Mean vessel density within 3-mm foveal region | 509 | 260 | -0.00137 (-0.00248, -0.00026) | -0.145 | 0.016 | 0.095 |
| Per 10 mL/min/1.73 m² lower eGFR | Mean vessel density within 5-mm foveal region | 509 | 260 | -0.00090 (-0.00191, 0.00012) | -0.096 | 0.083 | 0.095 |
| Per 10 mL/min/1.73 m² lower eGFR | Vascular fractal dimension | 509 | 260 | -0.00275 (-0.00564, 0.00015) | -0.101 | 0.063 | 0.095 |
| Per 10 mL/min/1.73 m² lower eGFR | Arterial fractal dimension | 509 | 260 | -0.00334 (-0.00677, 0.00008) | -0.105 | 0.056 | 0.095 |
| Per 10 mL/min/1.73 m² lower eGFR | Venous fractal dimension | 509 | 260 | -0.00209 (-0.00469, 0.00052) | -0.087 | 0.116 | 0.116 |

**Note.** Models adjusted for age, sex, BMI, diabetes duration, HbA1c, systolic blood pressure, and documented pre-admission insulin, ACEI/ARB, and statin use. FDR correction was applied separately by exposure.

**Supplementary Table S5. Sensitivity analysis with additional adjustment for eye-specific diabetic retinopathy stage.**

| **Exposure** | **Retinal phenotype** | **Eyes** | **Patients** | **β (95% CI)** | **Standardized β** | **P value** | **FDR q** |
| --- | --- | --- | --- | --- | --- | --- | --- |
| Per doubling of UACR | Vessel density | 485 | 248 | -0.00051 (-0.00145, 0.00044) | -0.060 | 0.292 | 0.604 |
| Per doubling of UACR | Arterial density | 485 | 248 | -0.00040 (-0.00085, 0.00004) | -0.100 | 0.076 | 0.604 |
| Per doubling of UACR | Venous density | 485 | 248 | -0.00010 (-0.00066, 0.00046) | -0.021 | 0.716 | 0.819 |
| Per doubling of UACR | Mean vessel density within 3-mm foveal region | 485 | 248 | -0.00033 (-0.00131, 0.00064) | -0.036 | 0.500 | 0.745 |
| Per doubling of UACR | Mean vessel density within 5-mm foveal region | 485 | 248 | -0.00050 (-0.00145, 0.00045) | -0.054 | 0.302 | 0.604 |
| Per doubling of UACR | Vascular fractal dimension | 485 | 248 | -0.00088 (-0.00386, 0.00209) | -0.034 | 0.559 | 0.745 |
| Per doubling of UACR | Arterial fractal dimension | 485 | 248 | -0.00197 (-0.00561, 0.00168) | -0.057 | 0.289 | 0.604 |
| Per doubling of UACR | Venous fractal dimension | 485 | 248 | -0.00005 (-0.00270, 0.00260) | -0.002 | 0.972 | 0.972 |
| Per 10 mL/min/1.73 m² lower eGFR | Vessel density | 509 | 260 | -0.00107 (-0.00204, -0.00011) | -0.125 | 0.029 | 0.076 |
| Per 10 mL/min/1.73 m² lower eGFR | Arterial density | 509 | 260 | -0.00049 (-0.00096, -0.00003) | -0.120 | 0.037 | 0.076 |
| Per 10 mL/min/1.73 m² lower eGFR | Venous density | 509 | 260 | -0.00058 (-0.00115, -0.00001) | -0.115 | 0.048 | 0.076 |
| Per 10 mL/min/1.73 m² lower eGFR | Mean vessel density within 3-mm foveal region | 509 | 260 | -0.00127 (-0.00233, -0.00021) | -0.135 | 0.019 | 0.076 |
| Per 10 mL/min/1.73 m² lower eGFR | Mean vessel density within 5-mm foveal region | 509 | 260 | -0.00086 (-0.00184, 0.00013) | -0.091 | 0.088 | 0.088 |
| Per 10 mL/min/1.73 m² lower eGFR | Vascular fractal dimension | 509 | 260 | -0.00280 (-0.00556, -0.00004) | -0.103 | 0.047 | 0.076 |
| Per 10 mL/min/1.73 m² lower eGFR | Arterial fractal dimension | 509 | 260 | -0.00314 (-0.00647, 0.00019) | -0.099 | 0.064 | 0.080 |
| Per 10 mL/min/1.73 m² lower eGFR | Venous fractal dimension | 509 | 260 | -0.00230 (-0.00479, 0.00019) | -0.096 | 0.070 | 0.080 |

**Note.** Models included the medication-expanded covariates and diabetic retinopathy stage of the corresponding eye. FDR correction was applied separately by exposure.

**Supplementary Table S6. Generalized estimating equation sensitivity analyses.**

| **Exposure** | **Retinal phenotype** | **Eyes** | **Patients** | **β (95% CI)** | **Standardized β** | **P value** | **FDR q** |
| --- | --- | --- | --- | --- | --- | --- | --- |
| Per doubling of UACR | Vessel density | 485 | 248 | -0.00050 (-0.00142, 0.00042) | -0.060 | 0.284 | 0.454 |
| Per doubling of UACR | Arterial density | 485 | 248 | -0.00050 (-0.00092, -0.00007) | -0.123 | 0.022 | 0.175 |
| Per doubling of UACR | Venous density | 485 | 248 | -0.00001 (-0.00056, 0.00054) | -0.002 | 0.968 | 0.968 |
| Per doubling of UACR | Mean vessel density within 3-mm foveal region | 485 | 248 | -0.00067 (-0.00166, 0.00032) | -0.073 | 0.185 | 0.454 |
| Per doubling of UACR | Mean vessel density within 5-mm foveal region | 485 | 248 | -0.00055 (-0.00149, 0.00038) | -0.060 | 0.245 | 0.454 |
| Per doubling of UACR | Vascular fractal dimension | 485 | 248 | -0.00091 (-0.00384, 0.00201) | -0.035 | 0.541 | 0.721 |
| Per doubling of UACR | Arterial fractal dimension | 485 | 248 | -0.00262 (-0.00597, 0.00073) | -0.075 | 0.125 | 0.454 |
| Per doubling of UACR | Venous fractal dimension | 485 | 248 | 0.00027 (-0.00241, 0.00295) | 0.012 | 0.843 | 0.963 |
| Per 10 mL/min/1.73 m² lower eGFR | Vessel density | 509 | 260 | -0.00092 (-0.00188, 0.00004) | -0.108 | 0.059 | 0.154 |
| Per 10 mL/min/1.73 m² lower eGFR | Arterial density | 509 | 260 | -0.00049 (-0.00094, -0.00004) | -0.119 | 0.034 | 0.135 |
| Per 10 mL/min/1.73 m² lower eGFR | Venous density | 509 | 260 | -0.00044 (-0.00102, 0.00014) | -0.088 | 0.135 | 0.155 |
| Per 10 mL/min/1.73 m² lower eGFR | Mean vessel density within 3-mm foveal region | 509 | 260 | -0.00127 (-0.00236, -0.00019) | -0.135 | 0.021 | 0.135 |
| Per 10 mL/min/1.73 m² lower eGFR | Mean vessel density within 5-mm foveal region | 509 | 260 | -0.00079 (-0.00178, 0.00020) | -0.085 | 0.116 | 0.154 |
| Per 10 mL/min/1.73 m² lower eGFR | Vascular fractal dimension | 509 | 260 | -0.00240 (-0.00525, 0.00045) | -0.089 | 0.099 | 0.154 |
| Per 10 mL/min/1.73 m² lower eGFR | Arterial fractal dimension | 509 | 260 | -0.00301 (-0.00635, 0.00034) | -0.095 | 0.078 | 0.154 |
| Per 10 mL/min/1.73 m² lower eGFR | Venous fractal dimension | 509 | 260 | -0.00177 (-0.00435, 0.00080) | -0.074 | 0.178 | 0.178 |

**Note.** GEE used a Gaussian family, identity link, exchangeable working correlation, and robust sandwich variance. Models adjusted for the core covariates. FDR correction was applied separately by exposure.

**Supplementary Table S7A. Single-exposure and mutually adjusted models in the same joint complete-case cohort.**

| **Retinal phenotype** | **UACR-only core model** | **eGFR-only core model** | **UACR coefficient in joint model** | **eGFR coefficient in joint model** |
| --- | --- | --- | --- | --- |
| Vessel density | -0.00049 (-0.00141, 0.00044); P=0.301; q=0.482 | -0.00134 (-0.00240, -0.00028); P=0.013; q=0.029 | -0.00022 (-0.00112, 0.00068); P=0.635; q=0.746 | -0.00128 (-0.00234, -0.00021); P=0.019; q=0.037 |
| Arterial density | -0.00046 (-0.00089, -0.00002); P=0.040; q=0.319 | -0.00065 (-0.00115, -0.00015); P=0.011; q=0.029 | -0.00034 (-0.00077, 0.00009); P=0.120; q=0.746 | -0.00055 (-0.00105, -0.00005); P=0.033; q=0.037 |
| Venous density | -0.00003 (-0.00059, 0.00053); P=0.917; q=0.917 | -0.00069 (-0.00132, -0.00006); P=0.032; q=0.042 | 0.00012 (-0.00042, 0.00067); P=0.653; q=0.746 | -0.00073 (-0.00136, -0.00010); P=0.024; q=0.037 |
| Mean vessel density within 3-mm foveal region | -0.00063 (-0.00166, 0.00040); P=0.228; q=0.482 | -0.00179 (-0.00300, -0.00058); P=0.004; q=0.029 | -0.00027 (-0.00127, 0.00073); P=0.598; q=0.746 | -0.00171 (-0.00298, -0.00044); P=0.008; q=0.037 |
| Mean vessel density within 5-mm foveal region | -0.00049 (-0.00143, 0.00044); P=0.299; q=0.482 | -0.00115 (-0.00223, -0.00006); P=0.038; q=0.044 | -0.00027 (-0.00121, 0.00067); P=0.573; q=0.746 | -0.00107 (-0.00219, 0.00006); P=0.064; q=0.064 |
| Vascular fractal dimension | -0.00100 (-0.00385, 0.00184); P=0.487; q=0.649 | -0.00365 (-0.00667, -0.00064); P=0.018; q=0.029 | -0.00025 (-0.00300, 0.00250); P=0.858; q=0.858 | -0.00358 (-0.00659, -0.00057); P=0.020; q=0.037 |
| Arterial fractal dimension | -0.00265 (-0.00602, 0.00072); P=0.123; q=0.482 | -0.00447 (-0.00804, -0.00090); P=0.014; q=0.029 | -0.00182 (-0.00509, 0.00144); P=0.273; q=0.746 | -0.00393 (-0.00742, -0.00044); P=0.028; q=0.037 |
| Venous fractal dimension | 0.00017 (-0.00240, 0.00275); P=0.894; q=0.917 | -0.00279 (-0.00552, -0.00006); P=0.045; q=0.045 | 0.00081 (-0.00169, 0.00331); P=0.522; q=0.746 | -0.00303 (-0.00576, -0.00030); P=0.030; q=0.037 |

**Note.** All models used the identical joint complete-case cohort of 431 eyes from 220 patients and adjusted for age, sex, BMI, diabetes duration, HbA1c, and systolic blood pressure. UACR was log2-transformed; eGFR was scaled per 10 mL/min/1.73 m² lower value. FDR correction was applied across the eight outcomes separately for each displayed exposure-model family. The eGFR-only model was FDR-significant for all eight outcomes, showing that the stronger signal in this cohort preceded mutual adjustment for UACR.

**Supplementary Table S7B. Medication-expanded joint model including UACR and 2021 CKD-EPI eGFR.**

| **Exposure** | **Retinal phenotype** | **Eyes** | **Patients** | **β (95% CI)** | **Standardized β** | **P value** | **FDR q** |
| --- | --- | --- | --- | --- | --- | --- | --- |
| Per doubling of UACR | Vessel density | 431 | 220 | -0.00023 (-0.00113, 0.00068) | -0.028 | 0.617 | 0.792 |
| Per doubling of UACR | Arterial density | 431 | 220 | -0.00034 (-0.00076, 0.00008) | -0.087 | 0.116 | 0.792 |
| Per doubling of UACR | Venous density | 431 | 220 | 0.00011 (-0.00044, 0.00065) | 0.023 | 0.693 | 0.792 |
| Per doubling of UACR | Mean vessel density within 3-mm foveal region | 431 | 220 | -0.00028 (-0.00127, 0.00072) | -0.031 | 0.585 | 0.792 |
| Per doubling of UACR | Mean vessel density within 5-mm foveal region | 431 | 220 | -0.00028 (-0.00122, 0.00066) | -0.032 | 0.554 | 0.792 |
| Per doubling of UACR | Vascular fractal dimension | 431 | 220 | -0.00028 (-0.00306, 0.00250) | -0.011 | 0.843 | 0.843 |
| Per doubling of UACR | Arterial fractal dimension | 431 | 220 | -0.00183 (-0.00507, 0.00140) | -0.065 | 0.265 | 0.792 |
| Per doubling of UACR | Venous fractal dimension | 431 | 220 | 0.00077 (-0.00176, 0.00331) | 0.034 | 0.549 | 0.792 |
| Per 10 mL/min/1.73 m² lower eGFR | Vessel density | 431 | 220 | -0.00125 (-0.00233, -0.00016) | -0.150 | 0.024 | 0.047 |
| Per 10 mL/min/1.73 m² lower eGFR | Arterial density | 431 | 220 | -0.00053 (-0.00103, -0.00002) | -0.131 | 0.041 | 0.047 |
| Per 10 mL/min/1.73 m² lower eGFR | Venous density | 431 | 220 | -0.00072 (-0.00137, -0.00008) | -0.148 | 0.029 | 0.047 |
| Per 10 mL/min/1.73 m² lower eGFR | Mean vessel density within 3-mm foveal region | 431 | 220 | -0.00165 (-0.00292, -0.00039) | -0.181 | 0.011 | 0.047 |
| Per 10 mL/min/1.73 m² lower eGFR | Mean vessel density within 5-mm foveal region | 431 | 220 | -0.00105 (-0.00218, 0.00009) | -0.115 | 0.071 | 0.071 |
| Per 10 mL/min/1.73 m² lower eGFR | Vascular fractal dimension | 431 | 220 | -0.00349 (-0.00658, -0.00041) | -0.136 | 0.027 | 0.047 |
| Per 10 mL/min/1.73 m² lower eGFR | Arterial fractal dimension | 431 | 220 | -0.00379 (-0.00734, -0.00024) | -0.129 | 0.036 | 0.047 |
| Per 10 mL/min/1.73 m² lower eGFR | Venous fractal dimension | 431 | 220 | -0.00299 (-0.00579, -0.00018) | -0.129 | 0.037 | 0.047 |

**Note.** Both kidney markers were entered simultaneously. Models additionally adjusted for documented pre-admission insulin, ACEI/ARB, and statin use. FDR correction was applied separately for the UACR and eGFR coefficients.

**Supplementary Table S8. Sensitivity analysis using laboratory-reported Chinese-modified MDRD eGFR.**

| **Exposure** | **Retinal phenotype** | **Eyes** | **Patients** | **β (95% CI)** | **Standardized β** | **P value** | **FDR q** |
| --- | --- | --- | --- | --- | --- | --- | --- |
| Per 10 mL/min/1.73 m² lower laboratory-reported eGFR | Vessel density | 453 | 233 | -0.00059 (-0.00098, -0.00020) | -0.151 | 0.003 | 0.007 |
| Per 10 mL/min/1.73 m² lower laboratory-reported eGFR | Arterial density | 453 | 233 | -0.00029 (-0.00048, -0.00010) | -0.155 | 0.003 | 0.007 |
| Per 10 mL/min/1.73 m² lower laboratory-reported eGFR | Venous density | 453 | 233 | -0.00030 (-0.00053, -0.00007) | -0.132 | 0.010 | 0.012 |
| Per 10 mL/min/1.73 m² lower laboratory-reported eGFR | Mean vessel density within 3-mm foveal region | 453 | 233 | -0.00078 (-0.00125, -0.00032) | -0.184 | 0.001 | 0.007 |
| Per 10 mL/min/1.73 m² lower laboratory-reported eGFR | Mean vessel density within 5-mm foveal region | 453 | 233 | -0.00056 (-0.00095, -0.00018) | -0.134 | 0.004 | 0.007 |
| Per 10 mL/min/1.73 m² lower laboratory-reported eGFR | Vascular fractal dimension | 453 | 233 | -0.00171 (-0.00288, -0.00054) | -0.134 | 0.004 | 0.007 |
| Per 10 mL/min/1.73 m² lower laboratory-reported eGFR | Arterial fractal dimension | 453 | 233 | -0.00171 (-0.00322, -0.00020) | -0.101 | 0.026 | 0.026 |
| Per 10 mL/min/1.73 m² lower laboratory-reported eGFR | Venous fractal dimension | 453 | 233 | -0.00141 (-0.00246, -0.00037) | -0.126 | 0.008 | 0.011 |

**Note.** Models adjusted for the core clinical covariates and used patient-clustered robust standard errors.

**Supplementary Table S9. Sensitivity analysis restricted to 2021 CKD-EPI eGFR <120 mL/min/1.73 m².**

| **Exposure** | **Retinal phenotype** | **Eyes** | **Patients** | **β (95% CI)** | **Standardized β** | **P value** | **FDR q** |
| --- | --- | --- | --- | --- | --- | --- | --- |
| Per 10 mL/min/1.73 m² lower eGFR | Vessel density | 427 | 219 | -0.00134 (-0.00243, -0.00026) | -0.138 | 0.015 | 0.038 |
| Per 10 mL/min/1.73 m² lower eGFR | Arterial density | 427 | 219 | -0.00072 (-0.00123, -0.00021) | -0.155 | 0.006 | 0.025 |
| Per 10 mL/min/1.73 m² lower eGFR | Venous density | 427 | 219 | -0.00062 (-0.00127, 0.00003) | -0.109 | 0.060 | 0.069 |
| Per 10 mL/min/1.73 m² lower eGFR | Mean vessel density within 3-mm foveal region | 427 | 219 | -0.00174 (-0.00293, -0.00055) | -0.163 | 0.004 | 0.025 |
| Per 10 mL/min/1.73 m² lower eGFR | Mean vessel density within 5-mm foveal region | 427 | 219 | -0.00127 (-0.00243, -0.00010) | -0.119 | 0.033 | 0.044 |
| Per 10 mL/min/1.73 m² lower eGFR | Vascular fractal dimension | 427 | 219 | -0.00360 (-0.00685, -0.00035) | -0.113 | 0.030 | 0.044 |
| Per 10 mL/min/1.73 m² lower eGFR | Arterial fractal dimension | 427 | 219 | -0.00462 (-0.00846, -0.00077) | -0.124 | 0.019 | 0.038 |
| Per 10 mL/min/1.73 m² lower eGFR | Venous fractal dimension | 427 | 219 | -0.00261 (-0.00554, 0.00033) | -0.093 | 0.082 | 0.082 |

**Note.** Models adjusted for the core clinical covariates and used patient-clustered robust standard errors.

**Supplementary Table S10. eGFR category, interval-adjusted, and tighter temporal-window analyses.**

| **Analysis / exposure** | **Retinal phenotype** | **Eyes** | **Patients** | **β (95% CI)** | **Standardized β** | **P value** | **FDR q** |
| --- | --- | --- | --- | --- | --- | --- | --- |
| eGFR categories \| eGFR <90 vs 90-119 | Vessel density | 509 | 260 | -0.00627 (-0.01112, -0.00141) | -0.141 | 0.012 | 0.032 |
| eGFR categories \| eGFR ≥120 vs 90-119 | Vessel density | 509 | 260 | -0.00434 (-0.00939, 0.00070) | -0.091 | 0.091 | 0.146 |
| eGFR categories \| eGFR <90 vs 90-119 | Arterial density | 509 | 260 | -0.00326 (-0.00558, -0.00094) | -0.153 | 0.006 | 0.032 |
| eGFR categories \| eGFR ≥120 vs 90-119 | Arterial density | 509 | 260 | -0.00241 (-0.00482, 0.00000) | -0.106 | 0.050 | 0.118 |
| eGFR categories \| eGFR <90 vs 90-119 | Venous density | 509 | 260 | -0.00301 (-0.00589, -0.00013) | -0.115 | 0.040 | 0.046 |
| eGFR categories \| eGFR ≥120 vs 90-119 | Venous density | 509 | 260 | -0.00194 (-0.00494, 0.00107) | -0.069 | 0.206 | 0.206 |
| eGFR categories \| eGFR <90 vs 90-119 | Mean vessel density within 3-mm foveal region | 509 | 260 | -0.00692 (-0.01262, -0.00122) | -0.142 | 0.018 | 0.032 |
| eGFR categories \| eGFR ≥120 vs 90-119 | Mean vessel density within 3-mm foveal region | 509 | 260 | -0.00383 (-0.00971, 0.00204) | -0.073 | 0.200 | 0.206 |
| eGFR categories \| eGFR <90 vs 90-119 | Mean vessel density within 5-mm foveal region | 509 | 260 | -0.00657 (-0.01177, -0.00136) | -0.135 | 0.014 | 0.032 |
| eGFR categories \| eGFR ≥120 vs 90-119 | Mean vessel density within 5-mm foveal region | 509 | 260 | -0.00497 (-0.01012, 0.00019) | -0.096 | 0.059 | 0.118 |
| eGFR categories \| eGFR <90 vs 90-119 | Vascular fractal dimension | 509 | 260 | -0.01678 (-0.03231, -0.00125) | -0.120 | 0.034 | 0.046 |
| eGFR categories \| eGFR ≥120 vs 90-119 | Vascular fractal dimension | 509 | 260 | -0.01290 (-0.02624, 0.00044) | -0.086 | 0.058 | 0.118 |
| eGFR categories \| eGFR <90 vs 90-119 | Arterial fractal dimension | 509 | 260 | -0.02224 (-0.04091, -0.00356) | -0.135 | 0.020 | 0.032 |
| eGFR categories \| eGFR ≥120 vs 90-119 | Arterial fractal dimension | 509 | 260 | -0.01716 (-0.03242, -0.00190) | -0.098 | 0.028 | 0.118 |
| eGFR categories \| eGFR <90 vs 90-119 | Venous fractal dimension | 509 | 260 | -0.01216 (-0.02578, 0.00146) | -0.098 | 0.080 | 0.080 |
| eGFR categories \| eGFR ≥120 vs 90-119 | Venous fractal dimension | 509 | 260 | -0.00924 (-0.02144, 0.00296) | -0.070 | 0.137 | 0.183 |
| UACR + interval adjustment \| Per doubling of UACR | Vessel density | 485 | 248 | -0.00062 (-0.00150, 0.00026) | -0.074 | 0.167 | 0.267 |
| UACR + interval adjustment \| Per doubling of UACR | Arterial density | 485 | 248 | -0.00054 (-0.00096, -0.00012) | -0.133 | 0.012 | 0.098 |
| UACR + interval adjustment \| Per doubling of UACR | Venous density | 485 | 248 | -0.00008 (-0.00061, 0.00045) | -0.017 | 0.756 | 0.864 |
| UACR + interval adjustment \| Per doubling of UACR | Mean vessel density within 3-mm foveal region | 485 | 248 | -0.00078 (-0.00176, 0.00020) | -0.085 | 0.117 | 0.267 |
| UACR + interval adjustment \| Per doubling of UACR | Mean vessel density within 5-mm foveal region | 485 | 248 | -0.00069 (-0.00159, 0.00022) | -0.074 | 0.136 | 0.267 |
| UACR + interval adjustment \| Per doubling of UACR | Vascular fractal dimension | 485 | 248 | -0.00131 (-0.00402, 0.00140) | -0.050 | 0.343 | 0.458 |
| UACR + interval adjustment \| Per doubling of UACR | Arterial fractal dimension | 485 | 248 | -0.00291 (-0.00619, 0.00037) | -0.084 | 0.082 | 0.267 |
| UACR + interval adjustment \| Per doubling of UACR | Venous fractal dimension | 485 | 248 | -0.00010 (-0.00254, 0.00234) | -0.004 | 0.934 | 0.934 |
| eGFR + interval adjustment \| Per 10 mL/min/1.73 m² lower eGFR | Vessel density | 509 | 260 | -0.00103 (-0.00201, -0.00005) | -0.120 | 0.040 | 0.093 |
| eGFR + interval adjustment \| Per 10 mL/min/1.73 m² lower eGFR | Arterial density | 509 | 260 | -0.00053 (-0.00100, -0.00006) | -0.129 | 0.027 | 0.093 |
| eGFR + interval adjustment \| Per 10 mL/min/1.73 m² lower eGFR | Venous density | 509 | 260 | -0.00050 (-0.00109, 0.00009) | -0.099 | 0.096 | 0.110 |
| eGFR + interval adjustment \| Per 10 mL/min/1.73 m² lower eGFR | Mean vessel density within 3-mm foveal region | 509 | 260 | -0.00137 (-0.00248, -0.00025) | -0.145 | 0.016 | 0.093 |
| eGFR + interval adjustment \| Per 10 mL/min/1.73 m² lower eGFR | Mean vessel density within 5-mm foveal region | 509 | 260 | -0.00089 (-0.00190, 0.00011) | -0.095 | 0.080 | 0.107 |
| eGFR + interval adjustment \| Per 10 mL/min/1.73 m² lower eGFR | Vascular fractal dimension | 509 | 260 | -0.00273 (-0.00557, 0.00010) | -0.101 | 0.058 | 0.093 |
| eGFR + interval adjustment \| Per 10 mL/min/1.73 m² lower eGFR | Arterial fractal dimension | 509 | 260 | -0.00336 (-0.00674, 0.00002) | -0.106 | 0.052 | 0.093 |
| eGFR + interval adjustment \| Per 10 mL/min/1.73 m² lower eGFR | Venous fractal dimension | 509 | 260 | -0.00205 (-0.00460, 0.00050) | -0.086 | 0.114 | 0.114 |
| UACR, interval ≤3 days \| Per doubling of UACR | Vessel density | 475 | 242 | -0.00072 (-0.00162, 0.00019) | -0.086 | 0.120 | 0.192 |
| UACR, interval ≤3 days \| Per doubling of UACR | Arterial density | 475 | 242 | -0.00057 (-0.00100, -0.00013) | -0.141 | 0.010 | 0.083 |
| UACR, interval ≤3 days \| Per doubling of UACR | Venous density | 475 | 242 | -0.00015 (-0.00069, 0.00039) | -0.031 | 0.586 | 0.670 |
| UACR, interval ≤3 days \| Per doubling of UACR | Mean vessel density within 3-mm foveal region | 475 | 242 | -0.00090 (-0.00190, 0.00009) | -0.099 | 0.075 | 0.150 |
| UACR, interval ≤3 days \| Per doubling of UACR | Mean vessel density within 5-mm foveal region | 475 | 242 | -0.00083 (-0.00175, 0.00008) | -0.091 | 0.074 | 0.150 |
| UACR, interval ≤3 days \| Per doubling of UACR | Vascular fractal dimension | 475 | 242 | -0.00169 (-0.00443, 0.00106) | -0.068 | 0.228 | 0.304 |
| UACR, interval ≤3 days \| Per doubling of UACR | Arterial fractal dimension | 475 | 242 | -0.00327 (-0.00663, 0.00009) | -0.097 | 0.056 | 0.150 |
| UACR, interval ≤3 days \| Per doubling of UACR | Venous fractal dimension | 475 | 242 | -0.00046 (-0.00293, 0.00201) | -0.021 | 0.715 | 0.715 |
| eGFR, interval ≤3 days \| Per 10 mL/min/1.73 m² lower eGFR | Vessel density | 499 | 254 | -0.00101 (-0.00196, -0.00006) | -0.122 | 0.037 | 0.084 |
| eGFR, interval ≤3 days \| Per 10 mL/min/1.73 m² lower eGFR | Arterial density | 499 | 254 | -0.00050 (-0.00096, -0.00005) | -0.125 | 0.031 | 0.084 |
| eGFR, interval ≤3 days \| Per 10 mL/min/1.73 m² lower eGFR | Venous density | 499 | 254 | -0.00051 (-0.00109, 0.00006) | -0.106 | 0.079 | 0.091 |
| eGFR, interval ≤3 days \| Per 10 mL/min/1.73 m² lower eGFR | Mean vessel density within 3-mm foveal region | 499 | 254 | -0.00134 (-0.00246, -0.00022) | -0.145 | 0.019 | 0.084 |
| eGFR, interval ≤3 days \| Per 10 mL/min/1.73 m² lower eGFR | Mean vessel density within 5-mm foveal region | 499 | 254 | -0.00085 (-0.00184, 0.00013) | -0.093 | 0.090 | 0.091 |
| eGFR, interval ≤3 days \| Per 10 mL/min/1.73 m² lower eGFR | Vascular fractal dimension | 499 | 254 | -0.00271 (-0.00540, -0.00001) | -0.109 | 0.049 | 0.084 |
| eGFR, interval ≤3 days \| Per 10 mL/min/1.73 m² lower eGFR | Arterial fractal dimension | 499 | 254 | -0.00318 (-0.00640, 0.00004) | -0.107 | 0.053 | 0.084 |
| eGFR, interval ≤3 days \| Per 10 mL/min/1.73 m² lower eGFR | Venous fractal dimension | 499 | 254 | -0.00210 (-0.00454, 0.00033) | -0.095 | 0.091 | 0.091 |
| UACR, same day \| Per doubling of UACR | Vessel density | 214 | 110 | -0.00056 (-0.00178, 0.00066) | -0.069 | 0.361 | 0.675 |
| UACR, same day \| Per doubling of UACR | Arterial density | 214 | 110 | -0.00052 (-0.00114, 0.00011) | -0.130 | 0.103 | 0.675 |
| UACR, same day \| Per doubling of UACR | Venous density | 214 | 110 | -0.00005 (-0.00081, 0.00071) | -0.010 | 0.899 | 0.989 |
| UACR, same day \| Per doubling of UACR | Mean vessel density within 3-mm foveal region | 214 | 110 | -0.00064 (-0.00223, 0.00094) | -0.066 | 0.422 | 0.675 |
| UACR, same day \| Per doubling of UACR | Mean vessel density within 5-mm foveal region | 214 | 110 | -0.00073 (-0.00217, 0.00070) | -0.079 | 0.315 | 0.675 |
| UACR, same day \| Per doubling of UACR | Vascular fractal dimension | 214 | 110 | -0.00090 (-0.00436, 0.00255) | -0.038 | 0.605 | 0.806 |
| UACR, same day \| Per doubling of UACR | Arterial fractal dimension | 214 | 110 | -0.00210 (-0.00665, 0.00246) | -0.072 | 0.364 | 0.675 |
| UACR, same day \| Per doubling of UACR | Venous fractal dimension | 214 | 110 | -0.00002 (-0.00305, 0.00301) | -0.001 | 0.989 | 0.989 |
| eGFR, same day \| Per 10 mL/min/1.73 m² lower eGFR | Vessel density | 213 | 109 | -0.00035 (-0.00174, 0.00104) | -0.042 | 0.619 | 0.830 |
| eGFR, same day \| Per 10 mL/min/1.73 m² lower eGFR | Arterial density | 213 | 109 | -0.00027 (-0.00100, 0.00045) | -0.067 | 0.458 | 0.830 |
| eGFR, same day \| Per 10 mL/min/1.73 m² lower eGFR | Venous density | 213 | 109 | -0.00008 (-0.00089, 0.00074) | -0.016 | 0.851 | 0.851 |
| eGFR, same day \| Per 10 mL/min/1.73 m² lower eGFR | Mean vessel density within 3-mm foveal region | 213 | 109 | -0.00060 (-0.00230, 0.00111) | -0.058 | 0.490 | 0.830 |
| eGFR, same day \| Per 10 mL/min/1.73 m² lower eGFR | Mean vessel density within 5-mm foveal region | 213 | 109 | -0.00028 (-0.00199, 0.00142) | -0.030 | 0.744 | 0.850 |
| eGFR, same day \| Per 10 mL/min/1.73 m² lower eGFR | Vascular fractal dimension | 213 | 109 | -0.00152 (-0.00538, 0.00234) | -0.063 | 0.437 | 0.830 |
| eGFR, same day \| Per 10 mL/min/1.73 m² lower eGFR | Arterial fractal dimension | 213 | 109 | -0.00236 (-0.00716, 0.00245) | -0.079 | 0.334 | 0.830 |
| eGFR, same day \| Per 10 mL/min/1.73 m² lower eGFR | Venous fractal dimension | 213 | 109 | -0.00086 (-0.00431, 0.00259) | -0.041 | 0.622 | 0.830 |

Note. Category models used eGFR 90–119 mL/min/1.73 m² as the reference. The <90, 90–119, and ≥120 mL/min/1.73 m² categories and the complementary eGFR <120 mL/min/1.73 m² restricted-range analysis were specified a priori before inspection of the study eGFR distribution or outcome results. The ≥120 category was intended as an analytical high-eGFR stratum, not as a diagnostic definition of hyperfiltration. Fixed thresholds around 120 mL/min/1.73 m² have been used in diabetes hyperfiltration research, but definitions vary with age, sex, population, and GFR ascertainment (main manuscript references 38–40). Interval-adjusted models added the absolute laboratory-image interval to the core covariates. Tighter-window analyses retained records with an interval ≤3 days or the same calendar day. FDR correction was applied within each analysis-exposure family.

**Supplementary Table S11. Restricted cubic spline tests for nonlinearity of 2021 CKD-EPI eGFR associations.**

| **Retinal phenotype** | **Eyes** | **Patients** | **P for nonlinearity** | **FDR q** |
| --- | --- | --- | --- | --- |
| Vessel density | 509 | 260 | 0.078 | 0.104 |
| Arterial density | 509 | 260 | 0.031 | 0.064 |
| Venous density | 509 | 260 | 0.307 | 0.307 |
| Mean vessel density within 3-mm foveal region | 509 | 260 | 0.023 | 0.064 |
| Mean vessel density within 5-mm foveal region | 509 | 260 | 0.005 | 0.039 |
| Vascular fractal dimension | 509 | 260 | 0.049 | 0.078 |
| Arterial fractal dimension | 509 | 260 | 0.032 | 0.064 |
| Venous fractal dimension | 509 | 260 | 0.110 | 0.126 |

**Note.** Knots were placed at the 5th, 35th, 65th, and 95th percentiles (60.9, 99.5, 111.3, 128.1 mL/min/1.73 m²). Models adjusted for the core covariates and used patient-clustered robust standard errors. Benjamini-Hochberg correction was applied across the eight nonlinearity tests.

**Supplementary Table S12. Kidney-marker availability and complete-case comparisons.**
(Patient-level comparisons; standardized mean differences are descriptive. Exact UACR unavailable includes absent and inequality-only results.)

S12A. Exact numeric UACR available versus unavailable

| **Characteristic** | **Included (n=281)** | **Excluded (n=56)** | **Standardized mean difference** |
| --- | --- | --- | --- |
| Age, years | 55.00 (46.00, 64.00) | 55.00 (44.75, 62.00) | 0.163 |
| Female sex | 80/281 (28.5) | 24/56 (42.9) | -0.300 |
| BMI, kg/m² | 23.53 (21.37, 25.68) | 23.03 (21.62, 26.55) | -0.021 |
| Diabetes duration, years | 3.00 (0.33, 10.00) | 3.00 (0.50, 10.00) | -0.006 |
| Systolic blood pressure, mmHg | 131.00 (121.00, 142.00) | 133.50 (121.75, 143.50) | 0.021 |
| HbA1c, % | 10.90 (8.40, 12.45) | 9.90 (8.05, 11.70) | 0.068 |
| DR stage (worse eye) | 0.00 (0.00, 1.00) | 0.00 (0.00, 1.00) | -0.013 |
| Any DR | 102/281 (36.3) | 16/56 (28.6) | 0.165 |

S12B. Exact-UACR core complete case versus excluded

| **Characteristic** | **Included (n=248)** | **Excluded (n=33)** | **Standardized mean difference** |
| --- | --- | --- | --- |
| Age, years | 55.00 (47.00, 64.25) | 53.00 (45.00, 61.00) | 0.119 |
| Female sex | 72/248 (29.0) | 8/33 (24.2) | 0.108 |
| BMI, kg/m² | 23.56 (21.48, 25.60) | 22.73 (21.02, 26.34) | 0.169 |
| Diabetes duration, years | 2.00 (0.40, 10.00) | 4.00 (0.17, 7.00) | -0.146 |
| Systolic blood pressure, mmHg | 132.00 (124.00, 143.00) | 123.00 (113.00, 132.00) | 0.277 |
| HbA1c, % | 10.90 (8.40, 12.50) | 11.00 (8.45, 11.65) | 0.284 |
| DR stage (worse eye) | 0.00 (0.00, 1.00) | 0.00 (0.00, 1.00) | 0.415 |
| Any DR | 93/248 (37.5) | 9/33 (27.3) | 0.219 |

S12C. 2021 CKD-EPI eGFR available versus unavailable

| **Characteristic** | **Included (n=276)** | **Excluded (n=61)** | **Standardized mean difference** |
| --- | --- | --- | --- |
| Age, years | 55.00 (46.00, 63.00) | 55.00 (45.00, 62.00) | -0.033 |
| Female sex | 80/276 (29.0) | 24/61 (39.3) | -0.218 |
| BMI, kg/m² | 23.56 (21.53, 25.70) | 22.77 (21.00, 26.40) | 0.166 |
| Diabetes duration, years | 3.00 (0.50, 10.00) | 2.00 (0.17, 10.00) | -0.018 |
| Systolic blood pressure, mmHg | 131.00 (120.00, 142.00) | 134.00 (123.00, 142.00) | 0.029 |
| HbA1c, % | 10.80 (8.40, 12.40) | 10.75 (7.72, 12.38) | 0.099 |
| DR stage (worse eye) | 0.00 (0.00, 1.00) | 0.00 (0.00, 0.00) | 0.309 |
| Any DR | 103/276 (37.3) | 15/61 (24.6) | 0.275 |

S12D. eGFR core complete case versus excluded

| **Characteristic** | **Included (n=260)** | **Excluded (n=16)** | **Standardized mean difference** |
| --- | --- | --- | --- |
| Age, years | 55.00 (47.00, 63.25) | 55.50 (37.75, 58.25) | 0.262 |
| Female sex | 77/260 (29.6) | 3/16 (18.8) | 0.254 |
| BMI, kg/m² | 23.55 (21.57, 25.55) | 25.86 (21.44, 26.82) | -0.290 |
| Diabetes duration, years | 3.00 (0.50, 10.00) | 4.50 (0.21, 7.25) | -0.160 |
| Systolic blood pressure, mmHg | 131.00 (122.00, 143.00) | 119.00 (111.25, 126.75) | 0.372 |
| HbA1c, % | 10.80 (8.47, 12.43) | 9.30 (7.17, 11.32) | 0.498 |
| DR stage (worse eye) | 0.00 (0.00, 1.00) | 0.00 (0.00, 1.00) | 0.166 |
| Any DR | 96/260 (36.9) | 7/16 (43.8) | -0.139 |

**Supplementary Table S12E. Joint core complete-case cohort versus excluded patients.**

| **Characteristic** | **Joint core complete case (n=220)** | **Excluded from joint analysis (n=117)** | **Standardized mean difference** |
| --- | --- | --- | --- |
| Age, years | 55.00 (46.75, 64.00) | 55.00 (45.00, 62.00) | 0.044 |
| Female sex, n (%) | 60/220 (27.3) | 44/117 (37.6) | -0.222 |
| BMI, kg/m² | 23.65 (21.63, 25.65) | 22.99 (21.00, 26.40)* | 0.108 |
| Diabetes duration, years | 2.00 (0.50, 10.00) | 3.00 (0.25, 10.00) | -0.038 |
| Systolic blood pressure, mmHg | 131.00 (123.00, 142.25) | 131.00 (120.00, 142.00) | 0.088 |
| HbA1c, % | 10.85 (8.48, 12.43) | 10.35 (8.00, 12.38)† | 0.053 |
| DR stage (worse eye) | 0.00 (0.00, 1.00) | 0.00 (0.00, 1.00) | 0.153 |
| Any diabetic retinopathy, n (%) | 83/220 (37.7) | 35/117 (29.9) | 0.166 |
| Documented smoking history, n (%) | 92/220 (41.8) | 34/117 (29.1) | 0.269 |

Note. Values are median (IQR) unless otherwise indicated. Standardized mean differences are descriptive; positive values indicate higher values or prevalence in the joint complete-case cohort. The joint core complete-case cohort required exact numeric UACR, 2021 CKD-EPI eGFR, and complete age, sex, BMI, diabetes duration, HbA1c, and systolic blood pressure. *BMI was available for 113/117 excluded patients. †HbA1c was available for 78/117 excluded patients. Smoking history was available for all 337 patients in the final retinal cohort.

**Supplementary Table S13. Operational definitions of automatically quantified retinal phenotypes.**

| **Phenotype** | **Input representation** | **Analysis region** | **Operational definition** | **Unit** |
| --- | --- | --- | --- | --- |
| Overall vessel density | Binary overall-vessel segmentation map | Full predefined retinal region; optic disc retained | Vessel pixels divided by valid pixels within the fixed overall analysis mask | Proportion |
| Arterial density | Binary artery-classified map | Fixed metric-specific region | Arterial vessel pixels divided by valid pixels in the corresponding software-defined mask; optic-disc exclusion followed the metric-specific mask | Proportion |
| Venous density | Binary vein-classified map | Fixed metric-specific region | Venous vessel pixels divided by valid pixels in the corresponding software-defined mask; optic-disc exclusion followed the metric-specific mask | Proportion |
| 3-mm foveal vessel density | Binary vessel map | Circle of 3-mm diameter centered on AI-localized fovea | Vessel pixels divided by valid pixels within the 3-mm circle | Proportion |
| 5-mm foveal vessel density | Binary vessel map | Circle of 5-mm diameter centered on AI-localized fovea | Vessel pixels divided by valid pixels within the 5-mm circle | Proportion |
| Overall fractal dimension | Binary overall-vessel map | Fixed vascular analysis region | Slope of ln[N(ε)] versus ln(1/ε) across predefined box sizes | Dimensionless |
| Arterial fractal dimension | Binary artery-classified map | Same metric-specific vascular region | Box-counting slope calculated from the arterial binary map | Dimensionless |
| Venous fractal dimension | Binary vein-classified map | Same metric-specific vascular region | Box-counting slope calculated from the venous binary map | Dimensionless |
| Microaneurysm and exudate counts | Automated lesion masks | Effective retinal region | Number of discrete AI-detected lesion objects | Count |
| Hemorrhage and exudate areas | Automated lesion masks | Effective retinal region | Lesion pixels converted to mm² using image-specific pixel-pitch calibration | mm² |

Note. All images were analyzed with EVisionAI version 4.0. The software used locked metric-specific numerator and denominator masks. For metrics designated as non-disc regional measurements, the optic disc was excluded from both numerator and denominator; overall vessel density retained the optic disc. Published EVisionAI-related literature provides supporting information on the platform family, but the exact version 4.0 artery-vein classification, foveal localization, vessel-density repeatability, fractal-dimension repeatability, and performance across diabetic-retinopathy severity were not independently validated in the present cohort. For all 658 eyes in the final analytical cohort, vessel segmentation, artery-vein classification, foveal-center localization, and derivation of the final vascular measurements were fully automated; no image-analysis outputs were manually edited or corrected before statistical analysis (0/658 eyes underwent manual modification).

**Supplementary Table S14. Post hoc smoking-adjusted sensitivity analyses.**

| **Analysis / exposure** | **Retinal phenotype** | **Eyes** | **Patients** | **β (95% CI)** | **P value** | **FDR q** |
| --- | --- | --- | --- | --- | --- | --- |
| Smoking-adjusted UACR core model | Vessel density | 485 | 248 | -0.00076 (-0.00164, 0.00012) | 0.092 | 0.146 |
| Smoking-adjusted UACR core model | Arterial density | 485 | 248 | -0.00059 (-0.00101, -0.00017) | 0.006 | 0.048 |
| Smoking-adjusted UACR core model | Venous density | 485 | 248 | -0.00017 (-0.00070, 0.00036) | 0.529 | 0.604 |
| Smoking-adjusted UACR core model | Mean vessel density within 3-mm foveal region | 485 | 248 | -0.00092 (-0.00191, 0.00006) | 0.066 | 0.146 |
| Smoking-adjusted UACR core model | Mean vessel density within 5-mm foveal region | 485 | 248 | -0.00079 (-0.00170, 0.00011) | 0.086 | 0.146 |
| Smoking-adjusted UACR core model | Vascular fractal dimension | 485 | 248 | -0.00163 (-0.00432, 0.00107) | 0.237 | 0.316 |
| Smoking-adjusted UACR core model | Arterial fractal dimension | 485 | 248 | -0.00310 (-0.00644, 0.00024) | 0.069 | 0.146 |
| Smoking-adjusted UACR core model | Venous fractal dimension | 485 | 248 | -0.00045 (-0.00283, 0.00194) | 0.714 | 0.714 |
| Same-joint-cohort eGFR-only + smoking | Vessel density | 431 | 220 | -0.00122 (-0.00226, -0.00017) | 0.023 | 0.044 |
| Same-joint-cohort eGFR-only + smoking | Arterial density | 431 | 220 | -0.00060 (-0.00111, -0.00010) | 0.019 | 0.044 |
| Same-joint-cohort eGFR-only + smoking | Venous density | 431 | 220 | -0.00061 (-0.00123, 0.00000) | 0.050 | 0.062 |
| Same-joint-cohort eGFR-only + smoking | Mean vessel density within 3-mm foveal region | 431 | 220 | -0.00168 (-0.00289, -0.00048) | 0.006 | 0.044 |
| Same-joint-cohort eGFR-only + smoking | Mean vessel density within 5-mm foveal region | 431 | 220 | -0.00106 (-0.00213, 0.00002) | 0.054 | 0.062 |
| Same-joint-cohort eGFR-only + smoking | Vascular fractal dimension | 431 | 220 | -0.00335 (-0.00633, -0.00037) | 0.028 | 0.044 |
| Same-joint-cohort eGFR-only + smoking | Arterial fractal dimension | 431 | 220 | -0.00424 (-0.00780, -0.00067) | 0.020 | 0.044 |
| Same-joint-cohort eGFR-only + smoking | Venous fractal dimension | 431 | 220 | -0.00245 (-0.00511, 0.00021) | 0.071 | 0.071 |
| Joint model: UACR coefficient + smoking | Vessel density | 431 | 220 | -0.00039 (-0.00128, 0.00051) | 0.401 | 0.669 |
| Joint model: UACR coefficient + smoking | Arterial density | 431 | 220 | -0.00041 (-0.00084, 0.00003) | 0.066 | 0.530 |
| Joint model: UACR coefficient + smoking | Venous density | 431 | 220 | 0.00002 (-0.00051, 0.00056) | 0.936 | 0.936 |
| Joint model: UACR coefficient + smoking | Mean vessel density within 3-mm foveal region | 431 | 220 | -0.00042 (-0.00143, 0.00059) | 0.418 | 0.669 |
| Joint model: UACR coefficient + smoking | Mean vessel density within 5-mm foveal region | 431 | 220 | -0.00040 (-0.00135, 0.00055) | 0.411 | 0.669 |
| Joint model: UACR coefficient + smoking | Vascular fractal dimension | 431 | 220 | -0.00066 (-0.00337, 0.00205) | 0.634 | 0.845 |
| Joint model: UACR coefficient + smoking | Arterial fractal dimension | 431 | 220 | -0.00217 (-0.00543, 0.00109) | 0.192 | 0.669 |
| Joint model: UACR coefficient + smoking | Venous fractal dimension | 431 | 220 | 0.00038 (-0.00204, 0.00280) | 0.758 | 0.866 |
| Joint model: eGFR coefficient + smoking | Vessel density | 431 | 220 | -0.00110 (-0.00215, -0.00005) | 0.041 | 0.074 |
| Joint model: eGFR coefficient + smoking | Arterial density | 431 | 220 | -0.00048 (-0.00098, 0.00003) | 0.065 | 0.074 |
| Joint model: eGFR coefficient + smoking | Venous density | 431 | 220 | -0.00062 (-0.00124, -0.00001) | 0.048 | 0.074 |
| Joint model: eGFR coefficient + smoking | Mean vessel density within 3-mm foveal region | 431 | 220 | -0.00155 (-0.00280, -0.00030) | 0.015 | 0.074 |
| Joint model: eGFR coefficient + smoking | Mean vessel density within 5-mm foveal region | 431 | 220 | -0.00093 (-0.00205, 0.00019) | 0.104 | 0.104 |
| Joint model: eGFR coefficient + smoking | Vascular fractal dimension | 431 | 220 | -0.00314 (-0.00609, -0.00019) | 0.037 | 0.074 |
| Joint model: eGFR coefficient + smoking | Arterial fractal dimension | 431 | 220 | -0.00355 (-0.00702, -0.00009) | 0.044 | 0.074 |
| Joint model: eGFR coefficient + smoking | Venous fractal dimension | 431 | 220 | -0.00257 (-0.00522, 0.00008) | 0.057 | 0.074 |

Note. These analyses were post hoc and were undertaken after raw-data audit identified smoking history as complete in the final cohort and imbalanced between the joint complete-case cohort and excluded patients. UACR was log2-transformed; eGFR coefficients are per 10 mL/min/1.73 m² lower value. All models included the core covariates (age, sex, BMI, diabetes duration, HbA1c, and systolic blood pressure) plus documented smoking history. The same-joint-cohort eGFR-only models used the identical 431-eye/220-patient sample as the joint model. FDR correction was applied across the eight outcomes separately within each displayed exposure-model family. These analyses are exploratory and do not replace the prespecified primary models.

**Supplementary Figure S1. Box-counting calculation of retinal vascular fractal dimension.**

A binary retinal vessel segmentation map was overlaid with grids of progressively decreasing box size (ε). At each scale, N(ε) was defined as the number of boxes containing at least one vessel pixel. Fractal dimension was estimated from the slope of the linear regression of ln[N(ε)] against ln(1/ε). Different grid colors distinguish adjacent boxes and do not denote artery-vein classification. The displayed fitted value is a methodological illustration.
